# MaNCP1, a C2H2 Zinc Finger Protein, Governs the Conidiation Pattern Shift through Regulating the Reductive Pathway for Nitric Oxide Synthesis in the Filamentous Fungus Metarhizium *acridum*

**DOI:** 10.1128/spectrum.00538-22

**Published:** 2022-05-10

**Authors:** Chaochuang Li, Dingxiang Xu, Meiwen Hu, Qipei Zhang, Yuxian Xia, Kai Jin

**Affiliations:** a Genetic Engineering Research Center, School of Life Sciences, Chongqing University, Chongqing, People’s Republic of China; b Chongqing Engineering Research Center for Fungal Insecticide, Chongqing, People’s Republic of China; c Key Laboratory of Gene Function and Regulation Technologies Under Chongqing Municipal Education Commission, Chongqing, People’s Republic of China; Université Côte d'Azur, CNRS, Inserm

**Keywords:** *Metarhizium acridum*, C2H2 zinc finger protein, MaNCP1, conidiation pattern, *MaNmrA*, nitric oxide

## Abstract

Asexual sporulation is the most common reproduction mode of fungi. Most filamentous fungi have two conidiation patterns, normal conidiation and microcycle conidiation, which may be regulated by nutritional conditions. Nitrogen source can affect the fungal conidiation pattern, but the regulatory mechanism is not fully understood. In this study, we report a C2H2 zinc finger protein, MaNCP1, which has typical transcription factor characteristics and is screened from the subtractive library regulated by nitrate in the entomopathogenic fungus Metarhizium acridum. MaNCP1 and its N-terminal play critical roles in the conidiation pattern shift. Further study shows that MaNCP1 interacts with *MaNmrA*, which also contributes to the conidiation pattern shift and is involved in the reductive pathway of nitric oxide (NO) synthesis. Intriguingly, the conidiation pattern of the *MaNCP1*-disruption strain (Δ*MaNCP1*) can be restored to microcycle conidiation when grown on the microcycle conidiation medium, SYA, supplemented with NO donor or overexpressing *MaNmrA* in Δ*MaNCP1*. Here, we reveal that MaNCP1 governs the conidiation pattern shift through regulating the reductive synthesis of NO by physically targeting *MaNmrA* in M. acridum. This work provides new mechanistic insights into how changes in nitrogen utilization are linked to the regulation of fungal morphological changes.

**IMPORTANCE** Fungal conidia play important roles in the response to environmental stimuli and evasion of the host immune system. The nitrogen source is one of the main factors affecting shifts in fungal conidiation patterns, but the regulatory mechanism involved is not fully understood. In this work, we report that the C2H2 zinc finger protein, MaNCP1, governs the conidiation pattern shift in M. acridum by targeting the *MaNmrA* gene, thereby altering the regulation of the reductive pathway for NO synthesis. This work provides further insights into how the nutritional environment can regulate the morphogenesis of filamentous fungi.

## INTRODUCTION

Entomopathogenic fungi, such as Metarhizium spp., are fungi that can infect and cause the death of their insect hosts; they play important roles in the control of agricultural pests (1). Conidia generated by asexual reproduction play an important role in the process of fungal distribution due to their resistance to adverse environmental conditions, host immune attack and proliferation ability (2–4). Moreover, conidia are the main infective vectors of entomopathogenic fungi (1) and the main effective unit of insecticidal fungal pesticides. The production of conidia is therefore a prerequisite for the large-scale industrial production of biocontrol fungi (1, 5–7).

Most filamentous fungi, including the model entomopathogenic fungus *M. acridum*, have two conidiation patterns: normal conidiation and microcycle conidiation (8). Furthermore, it has been shown that the microcycle conidiation exhibits a greater potential for use in biological pest control than normal conidiation in *M. acridum* (9). Normal conidiation occurs in the typical life cycle of filamentous fungi, which comprises conidiation after a period of vegetative growth (10). Microcycle conidiation bypasses the vegetative hyphae and produces conidia directly from the germinated conidia (8). Usually, shifting from the normal conidiation to the microcycle conidiation is a survival mechanism for fungi to adapt to adverse environmental factors, including temperature (10), pH (11), salt concentration (12) and nutrients (13). Nutrition is one of the most important influencing factors and the two conidiation patterns are interconvertible under different nutrition conditions in many fungi (14). Changing the content of nitrogen source (nitrate) in the microcycle conidiation medium, SYA, can shift the conidiation pattern of *M. acridum* to normal (15), although the underlying mechanism is still unclear. Therefore, elucidating how changes in nutrient nitrate conditions can drive shifts in the conidiation pattern should provide further insights into the regulatory roles of nitrogen utilization in fungal morphogenesis.

Nitrogen is necessary for the growth of fungi, which preferentially use nitrogen sources such as ammonium and glutamine, but can use alternative sources, such as nitrate, urea, guanidine and pyrimidine where ammonium and glutamine are absent (16). This nitrogen source selection, called nitrogen catabolite repression (NCR), is mainly mediated by AreA, a GATA transcription factor in A. nidulans (17). *NmrA*, a member of the short-chain dehydrogenase reductase superfamily, is a core regulator gene in the NCR pathway that affects fungal nitrogen metabolism (18, 19) by interacting with and inhibiting the activity of AreA (20). The expression level of *NmrA* increases with sufficient nitrogen availability but decreases under nitrogen starvation (19). *NmrA* is also regulated by the bZIP transcription factor MeaB (21), which can work with AreA synergistically or independently in the NCR pathway (19). Three NmrA homologous proteins, Nmr1, Nmr2 and Nmr3 have been characterized in Magnaporthe oryzae, which can bind to NADP^+^ and are regulated by the NADPH sensor protein Tps1 (22), which could trigger carbon catabolite repression (CCR) *via* the inactivation of Nmr1-3 in a mechanism independent of Nut1, an AreA homologous protein (23). In Aspergillus nidulans, NmrA, together with AreA and AreB (another GATA transcription factor), negatively regulate arginine catabolism in response to nitrogen and carbon source (24). Furthermore, NmrA appears to be involved in sclerotia formation, conidiation, virulence and aflatoxin biosynthesis in A. flavus (25).

Nitric oxide (NO), as a signaling molecule, plays important roles in many biological processes (26). NO can be produced *via* the reductive pathway, in which nitrate is reduced by nitrate reductase (NR), or the oxidative pathway, where L-arginine is oxidized by nitric oxide synthase (NOS) (26). However, no clear homologs to NOS have been identified in fungi where the NO production apparently mainly occurs *via* the reductive pathway (26, 27). NO can be detected in many filamentous fungi, such as Neurospora crassa (28), Phycomyces blakesleeanus (29), Colletotrichum coccodes (30), Blastocladiella emersonii (31), Coniothyrium minitans (32) and Fusarium graminearum (33). Previous studies have shown that exogenous NO can inhibit the light-induced conidiation in N. crassa (28) and A. nidulans (34). In C. minitans, NO is accumulated heavily during the formation of conidia (35) and regulates the conidiation through the cGMP signaling pathway (31, 35). However, the regulation of NO production during fungal conidiation remains largely uncharacterized.

To reveal the molecular components involved in transducing nitrate effects on the conidiation pattern in M. acridum, a differential expression library regulated by nitrate was constructed (15). A C2H2 zinc finger protein MAC_04326 with transcriptional factor activity was identified from this library and denominated MaNCP1 (M. acridum nitrate-related conidiation pattern shift regulatory factor 1). In M. oryzae, MGG_07339 (the homologue of *MaNCP1*) is involved in regulating the conidiation and virulence on rice seedlings (36). In this study, we found that MaNCP1 governs the conidiation pattern shift through regulating the reductive pathway for NO synthesis *via* physical interaction with the *MaNmrA* gene in M. acridum. These results provide insights into the regulatory molecular network linking changes in nitrate metabolism with shifts in conidiation patterns of filamentous fungi.

## RESULTS

### MaNCP1 contributes to the conidiation pattern shift.

To explore the regulatory roles of genes mediated by nitrate in regulating the conidiation pattern shift, eight putative transcriptional factor genes were screened from the differential expression library regulated by nitrate (15) and further verified by quantitative reverse-transcription PCR (qRT-PCR). The results showed that *MAC_02692*, *MAC_04326* and *MAC_03846* were specifically upregulated in SYA+N medium (Fig. S1A), which were consistent with the transcriptome data. However, no further evidence for a role for either *MAC_02692* or *MAC_03846* in the conidiation pattern shift was observed (Fig. S1B). In contrast, the deletion of *MAC_04326* (*MaNCP1*) to produce the strain Δ*MaNCP1* led to the normal conidiation of M. acridum, which could be restored to microcycle conidiation by reintroducing the *MaNCP1* cassette into Δ*MaNCP1* on SYA medium (Fig. S1C, and S2A-C). In addition, the deletion of *MaNCP1* resulted in an increasing number of septa (Fig. S3A and B), longer hyphae (Fig. S3A and C) and an abnormal distribution of Spitzenkörper in the hypha apex (arrows in Fig. S3D), indicating *MaNCP1* was involved in hyphal polar growth.

**FIG 1:**
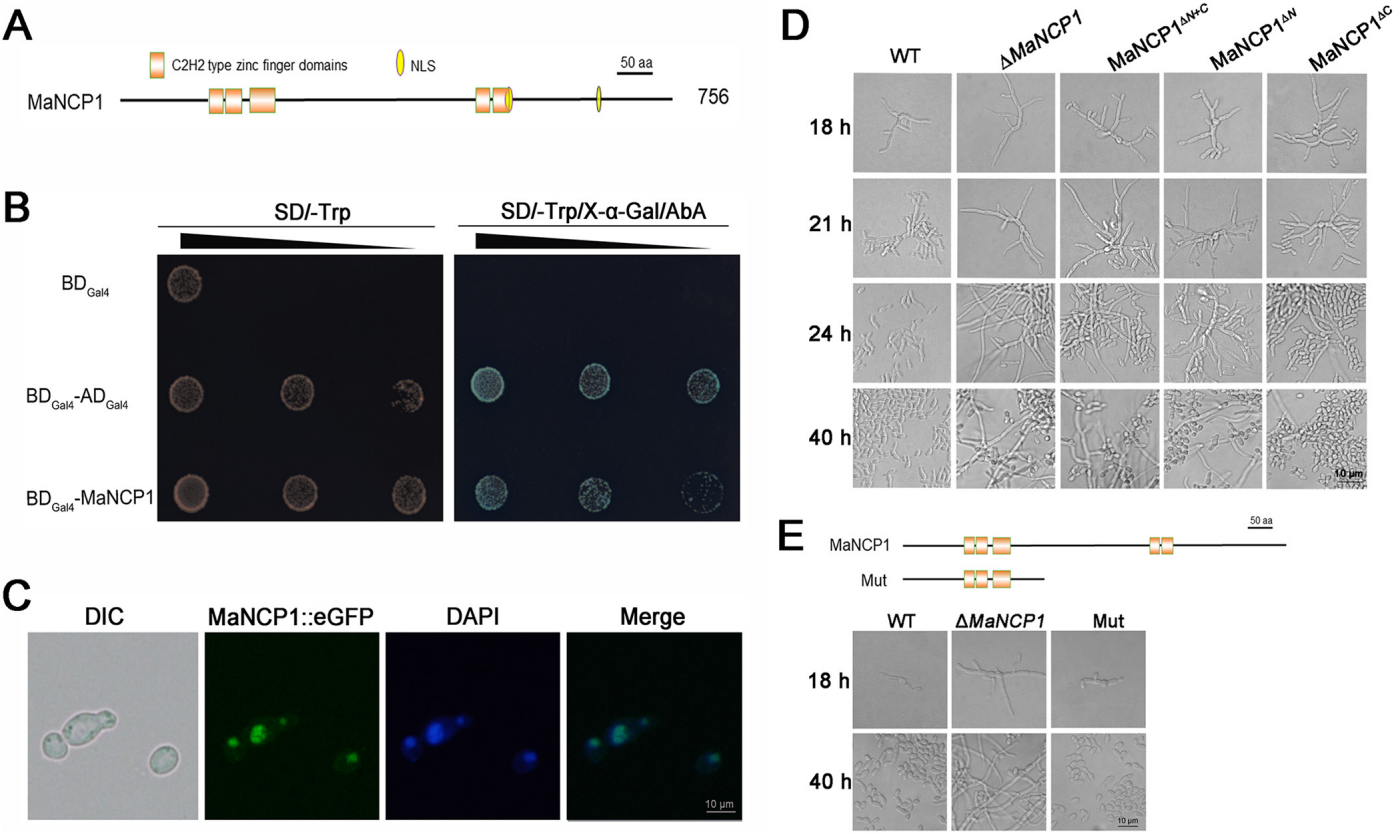
The N-terminal zinc fingers of MaNCP1 play important roles in the conidiation pattern shift. (A) Structural features of MaNCP1. (B) Autoactivation testing of MaNCP1 using the yeast two-hybrid assay. The empty vector, pGBKT7, was used as a negative control and pGBKT7-53 and pGADT7-T were used as a positive control. (C) MaNCP1::eGFP subcellular localization. (D) Conidiation patterns of the WT, Δ*MaNCP1*, MaNCP1^Δ^*^N^* (the N-terminal C2H2 zinc fingers deletion mutant), MaNCP1^Δ^*^C^* (The C-terminal C2H2 zinc fingers deletion mutant) and MaNCP1^Δ^*^N+C^* (The combined N- and C-terminal C2H2 zinc fingers deletion mutant) strains grown on SYA at 28°C for hours. (E) Conidiation pattern of the truncated protein Mut strain without the C-terminal of MaNCP1.

*MaNCP1* encodes a C2H2 zinc finger domain protein of 756 amino acid residues, with a predicted molecular mass of 84.95 kDa and an isoelectric point of 6.35. The MaNCP1 protein contains two predicted nuclear localization signals, three C2H2-type zinc fingers in the N-terminal and a further two C2H2-type zinc fingers in the C-terminal (Fig. 1A). The homologous zinc finger proteins are also found in other filamentous fungi (Fig. S4A) and the zinc fingers are highly similar to each other (Fig. S4B). Further phylogenetic analysis and multiple sequence alignments show that MaNCP1 is relatively close in evolution to filamentous fungi but far from yeast (Fig. S4C and S5). A transcriptional activity assay indicated that MaNCP1 has transcriptional activation ability (Fig. 1B). Furthermore, the MaNCP1::eGFP fusion protein locates in the nucleus (Fig. 1C). Thus, MaNCP1 has typical characteristics of a transcriptional factor.

To further observe the influence of the different zinc finger domains on the conidiation pattern shift, the domain deletion mutants MaNCP1^Δ^*^N^*, MaNCP1^Δ^*^C^*, and MaNCP1^Δ^*^N+C^* were obtained (Fig. S2D and E). It showed that the conidiation pattern of the MaNCP1^Δ^*^C^* strain was similar to the WT, while the MaNCP1^Δ^*^N^* strain was similar to the Δ*MaNCP1* when grown on SYA (Fig. 1D). In addition, the C-terminal truncated MaNCP1 strain (Mut) was constructed and exhibited microcycle conidiation on SYA, which was similar to the WT strain (Fig. 1E) and demonstrates a greater contribution of the N-terminal of MaNCP1 to the conidiation pattern shift than that of the C-terminal.

### *MaNmrA* is a direct target gene of MaNCP1 during the conidiation pattern shift.

To reveal the functions of *MaNCP1* in regulating the conidiation pattern shift, RNA-seq was performed to search the differentially expressed genes (DEGs) between the WT and Δ*MaNCP1* strains. Although the expression level of *MaNCP1* was highest at 21 h (Fig. S6A), the samples of Δ*MaNCP1* and WT strains were collected at 18 h for RNA-seq analysis, the earliest time point of observable difference in the conidiation pattern. A total of 9,552 genes were mapped to the *M. acridum* genome and there were 95 DEGs in Δ*MaNCP1*-*vs*-WT (Fig. S7A; Table S1), and these DEGs were involved in a variety of cellular processes and metabolic pathways (Fig. S7B and S7C). Furthermore, all the randomly selected DEGs exhibited a similar expression pattern to that observed in their respective DEG data (Fig. S6D), indicating that the RNA-seq data was reliable.

**FIG 2:**
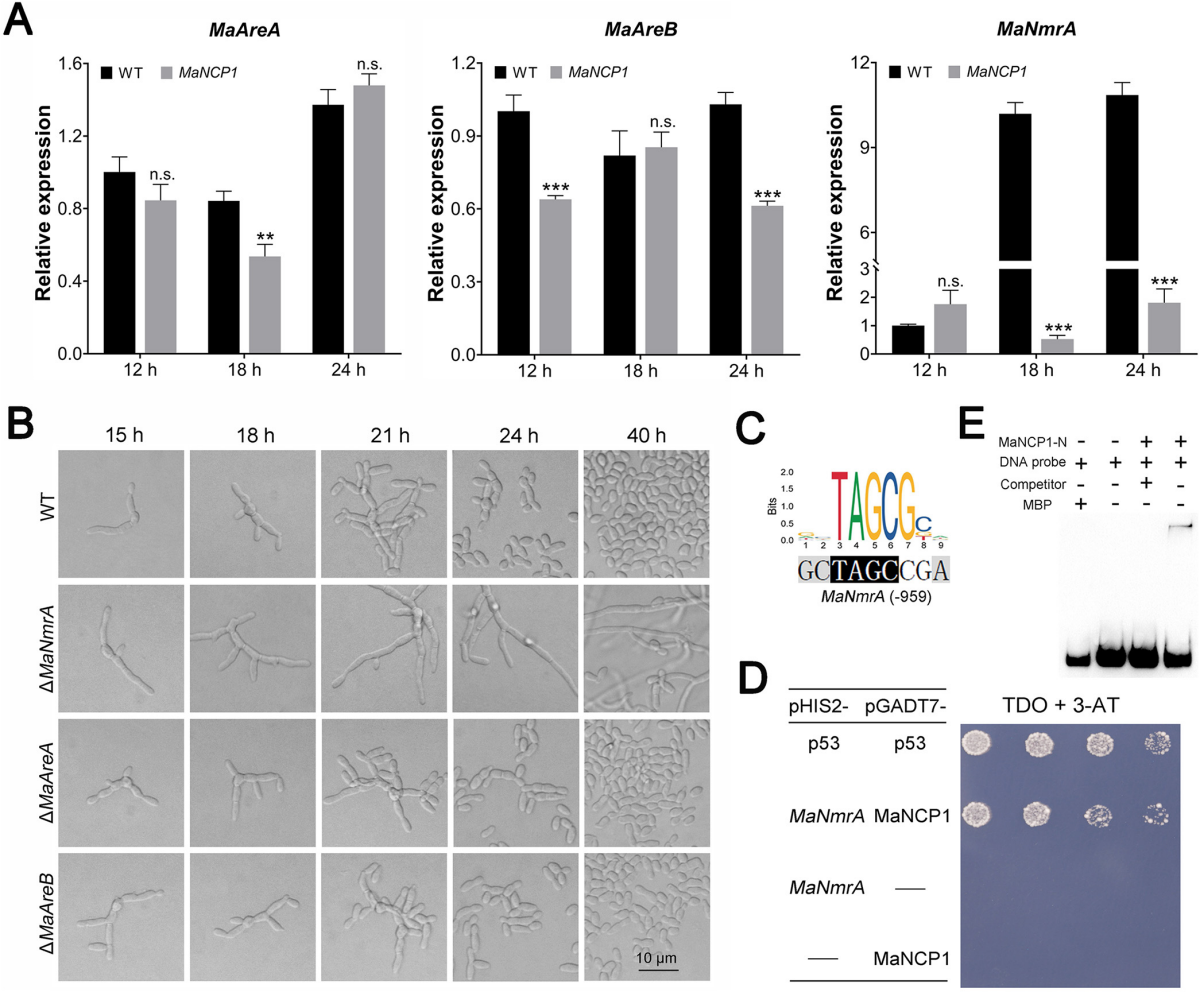
*MaNmrA* is a direct target gene of MaNCP1. (A) Relative expression analysis of *MaAreA*, *MaAreB* and *MaNmrA*. Fungal samples for qRT-PCR were collected at 12, 18 and 24 h of culture on SYA medium at 28°C. n.s. no significant difference, *P > *0.05. **, *P < *0.01; ***, *P < *0.001. (B) Conidiation pattern of the *MaAreA*, *MaAreB* and *MaNmrA* mutants grown on SYA media. (C) Prediction of the putative MaNCP1 binding site in *cis-elements* present in the promoters of potential downstream target genes of MaNCP1 were identified using the JASPAR 2020 database (54). (D) Yeast one-hybrid assay on TDO, SD/-Leu/-Trp/-His plates. 55 mM 3-AT was added into TDO plate. pHIS2-p53 and pGADT7-p53 acted as the positive control. pHIS2 and pGADT7-MaNCP1 or pGADT7 and pHIS2-*MaNmrA* acted as the negative control. (E) EMSA of MaNCP1 interaction with its putative binding site in *MaNmrA*. MaNCP1-N, the zinc finger cluster at the N-terminal of MaNCP1. MBP, the MBP-tag protein. Competitor, the unlabeled probe, was used in 100-fold excess to the biotin labeled probe.

Moreover, MaNCP1 was screened from the differential expression library regulated by nitrate, and *MAC_02196*, an NmrA family member gene, was selected from the RNA-seq DEG data to be characterized (Fig. S7D). Unfortunately, there was no obvious difference in conidiation pattern between the Δ*MAC_02196* and WT strains (Fig. S6B and C). Thus, we speculated whether other genes in the NCR pathway may be regulated by MaNCP1. Subsequently, three core genes of the NCR pathway of M. acridum, *MaAreA* (*MAC_00939*) (Fig. S8A and B), *MaAreB* (*MAC_07065*) (37) and *MaNmrA* (*MAC_00749*) (38) were selected for further analyses. A qRT-PCR analysis of the expression of these genes in Δ*MaNCP1* versus WT on SYA medium (Fig. 2A) showed that the whereas the expression of *MaAreA* in Δ*MaNCP1* was about 0.7 times of the WT at 18 h and *MaAreB* was about 0.8 times of the WT at 24 h, the expression of *MaNmrA* was reduced to ca. 0.05 times and 0.14 times of the WT at 18 h and 24 h, respectively. Furthermore, after the disruption of these three genes, only the conidiation pattern of the *MaNmrA*-disruption strain (Δ*MaNmrA*) was shifted to normal conidiation (Fig. 2B), similar to that observed in Δ*MaNCP1*. Moreover, the promoter of *MaNmrA* contained the putative binding site of MaNCP1 (Fig. 2C). Yeast one-hybrid assay showed that the recombinant yeast Y187 (pGADT7-MaNCP1×pHIS2-*MaNmrA*) could grow on TDO plates with the optimal concentration of 3-AT (55 mM) (Fig. S8C and Fig. 2D), indicating that *MaNmrA* was a direct target gene of MaNCP1. Moreover, the N-terminal of MaNCP1 (228 aa) was expressed in E. coli (Fig. S8D and E) and an electrophoretic mobility shift assay (EMSA) demonstrated that the N-terminal of MaNCP1 could specifically recognize the promoter of *MaNmrA* (Fig. 2E). Taken together, the data indicates that MaNCP1 may regulate the conidiation pattern by control of *MaNmrA*.

### *MaNCP1* and *MaNmrA* regulate the reductive pathway of NO synthesis.

**FIG 3:**
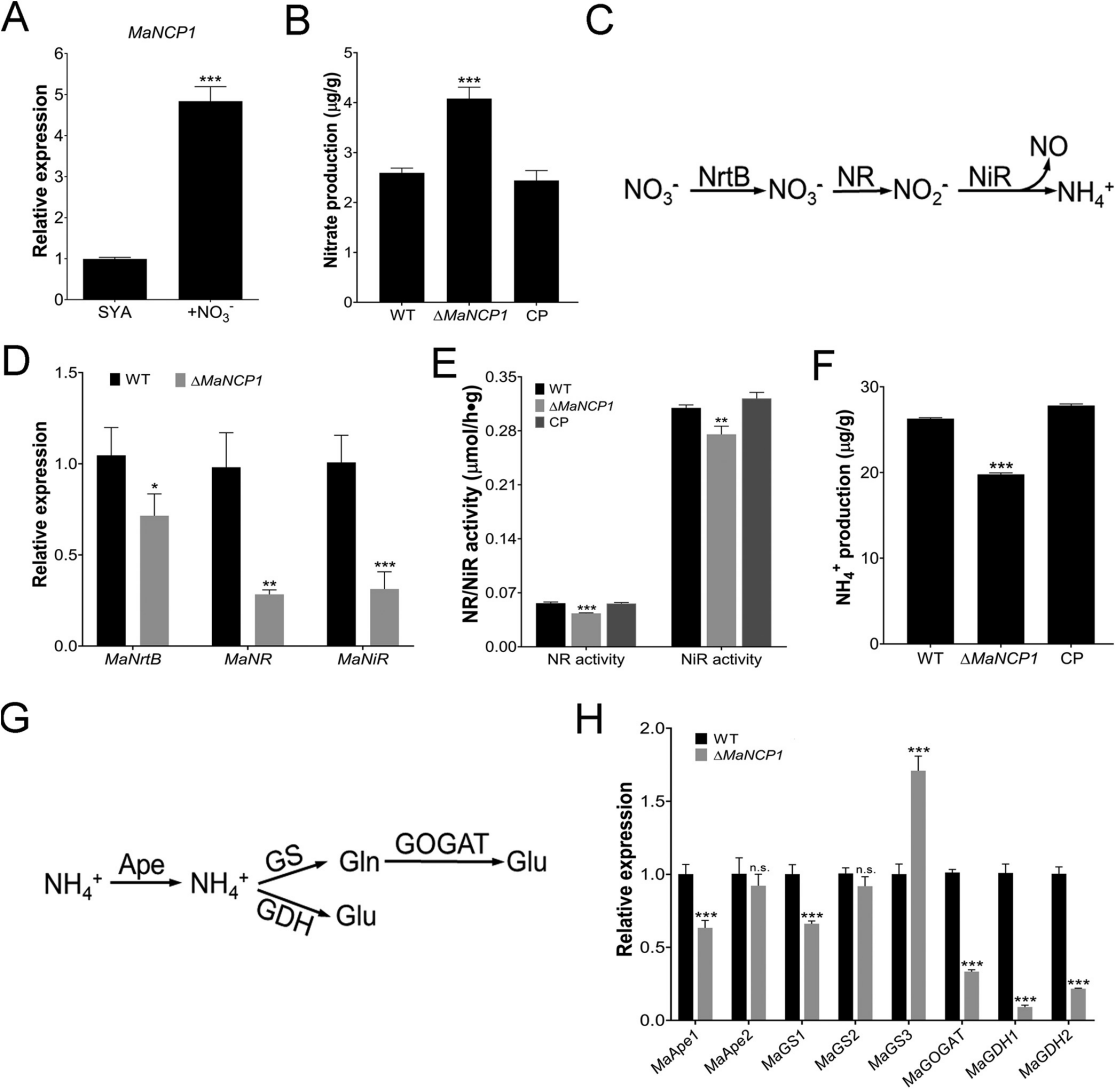
Deletion of *MaNCP1* affects the reductive pathway of nitrogen utilization and NO synthesis. (A) Relative expression of *MaNCP1* in WT strain under SYA and SYA+N conditions. (B) Determination of the nitrate content in the WT, Δ*MaNCP1* and CP strains. (C) Schematic diagram of the nitrate assimilation pathway. (D) Relative expression of *MaNrtB*, *MaNR* and *MaNiR* in the Δ*MaNCP1* background. (E) Determination of the NR and NiR activities in the WT, Δ*MaNCP1* and CP strains. (F) Determination of the ammonium content in the WT, Δ*MaNCP1* and CP strains. (G) Schematic diagram of the ammonium assimilation pathway. (H) Relative expression of genes related to ammonium assimilation in the Δ*MaNCP1* background. All fungal samples were grown on SYA at 28°C for 24 h. CP, the Δ*MaNCP1* complementary strain. n.s. no significant difference, *P > *0.05. *, *P < *0.05; **, *P < *0.01; ***, *P < *0.001.

**FIG 4:**
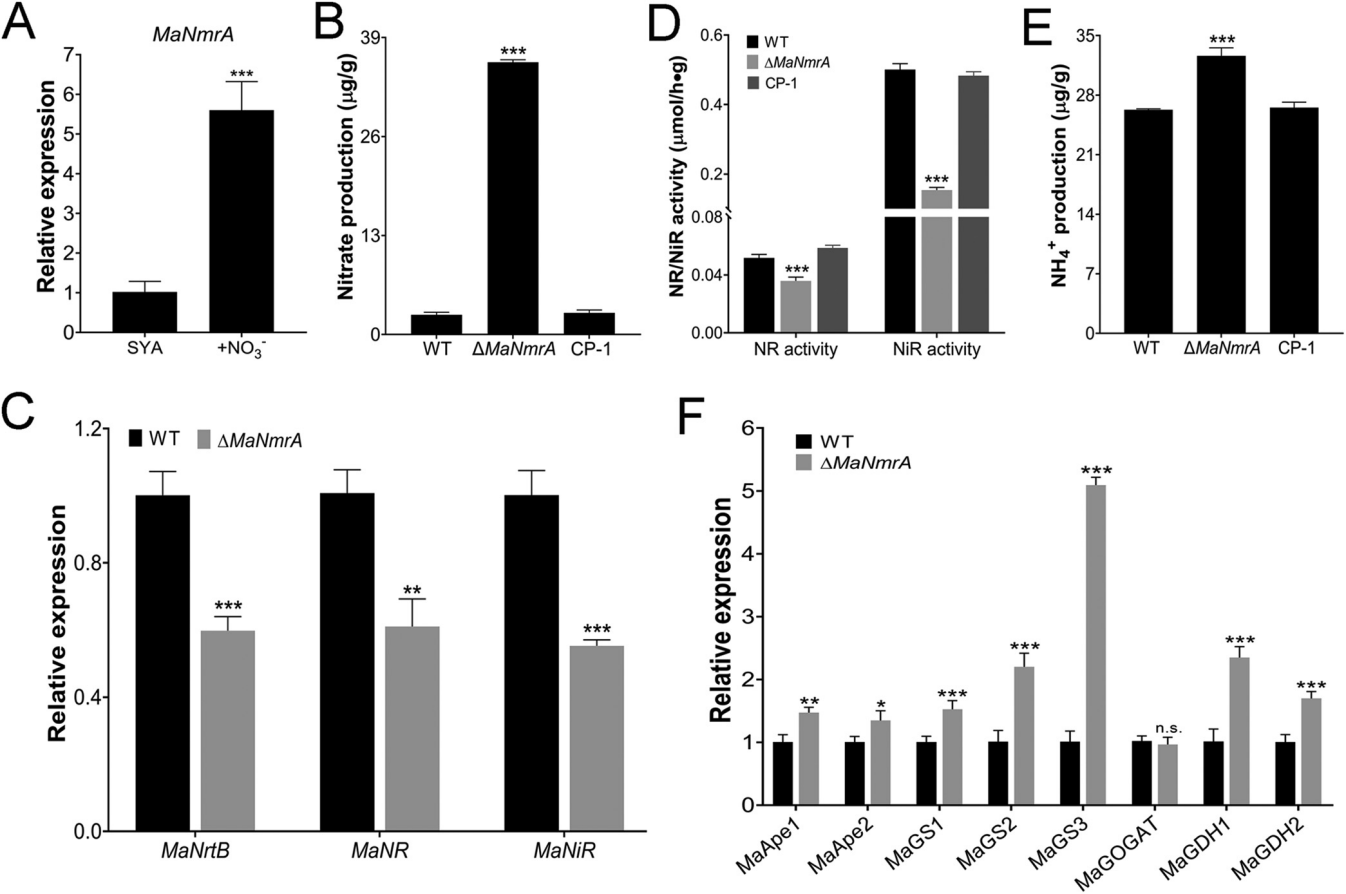
Deletion of *MaNmrA* affects the reductive pathway of NO synthesis and nitrogen utilization. (A) Relative expression of *MaNmrA* in the WT strain under SYA and SYA+N conditions. (B) Determination of the nitrate content in the WT, Δ*MaNmrA* and the Δ*MaNmrA* complementary (CP-1) strains. (C) Relative expression of *MaNrtB*, *MaNR* and *MaNiR* in the Δ*MaNmrA* background. (D) Determination of the NR and NiR activities in WT, Δ*MaNmrA* and CP-1 strains. (E) Determination of the ammonium content in WT, Δ*MaNmrA* and CP-1 strains. (F) Relative expression of genes related to ammonium assimilation in the Δ*MaNmrA* background. All fungal samples were grown on SYA at 28°C for 24 h. n.s. no significant difference, *P > *0.05. *, *P < *0.05; **, *P < *0.01; ***, *P < *0.001.

NmrA plays an important role in regulating the reductive pathway of NO synthesis, that is, the nitrate assimilation process (18, 19). Therefore, we tested whether *MaNCP1* also affected the reductive synthesis of NO. The transcription level of *MaNCP1* was significantly upregulated in SYA+N medium (Fig. 3A). The nitrate content in the Δ*MaNCP1* strain was significantly higher than that in the WT or CP strain (Fig. 3B). qRT-PCR analysis showed that the gene expression levels of the nitrate transporter, *MaNrtB* (*MAC_03189*), nitrate reductase, *MaNR* (*MAC_08624*) and nitrite reductase, *MaNiR* (*MAC_03493*), which play important roles in the process of nitrate assimilation (Fig. 3C), were significantly lower in Δ*MaNCP1* relative to the WT (Fig. 3D). Moreover, NR and NiR activities and the ammonium content in the Δ*MaNCP1* strain were significantly reduced (Fig. 3E and F). The expression of a series of genes functionally involved in the process of ammonium assimilation were subsequently analyzed. Whereas no significant difference was observed in the expression levels of *MaApe2* (*MAC_03001*) and *MaGS2* (*MAC_06858*), the genes *MaApe1* (*MAC_05564*), *MaGS1* (*MAC*_*01108*), *MaGOGAT* (*MAC*_*00032*), *MaGDH1* (*MAC_08384*) and *MaGDH2* (*MAC_01648*), were significantly downregulated, while *MaGS3* (*MAC_04461*) was significantly upregulated (Fig. 3G and H). Similarly, the expression of *MaNmrA* was also significantly upregulated in SYA+N media (Fig. 4A). Furthermore, the nitrate content in the Δ*MaNmrA* strain was higher than that in WT or CP-1 (the Δ*MaNmrA* complementary strain; Fig. 4B) and the expression levels of *MaNrtB*, *MaNR* and *MaNiR*, as well as the activities of NR and NiR in the Δ*MaNmrA* background were significantly reduced (Fig. 4C and D). However, the ammonium content in the Δ*MaNmrA* strain was higher (Fig. 4E) and genes involved in ammonium assimilation were also upregulated in the Δ*MaNmrA* background, except for *MaGOGAT*, which was not significantly different from that observed in the WT (Fig. 4F).

### NO is involved in the regulation of conidiation pattern shift.

**FIG 5:**
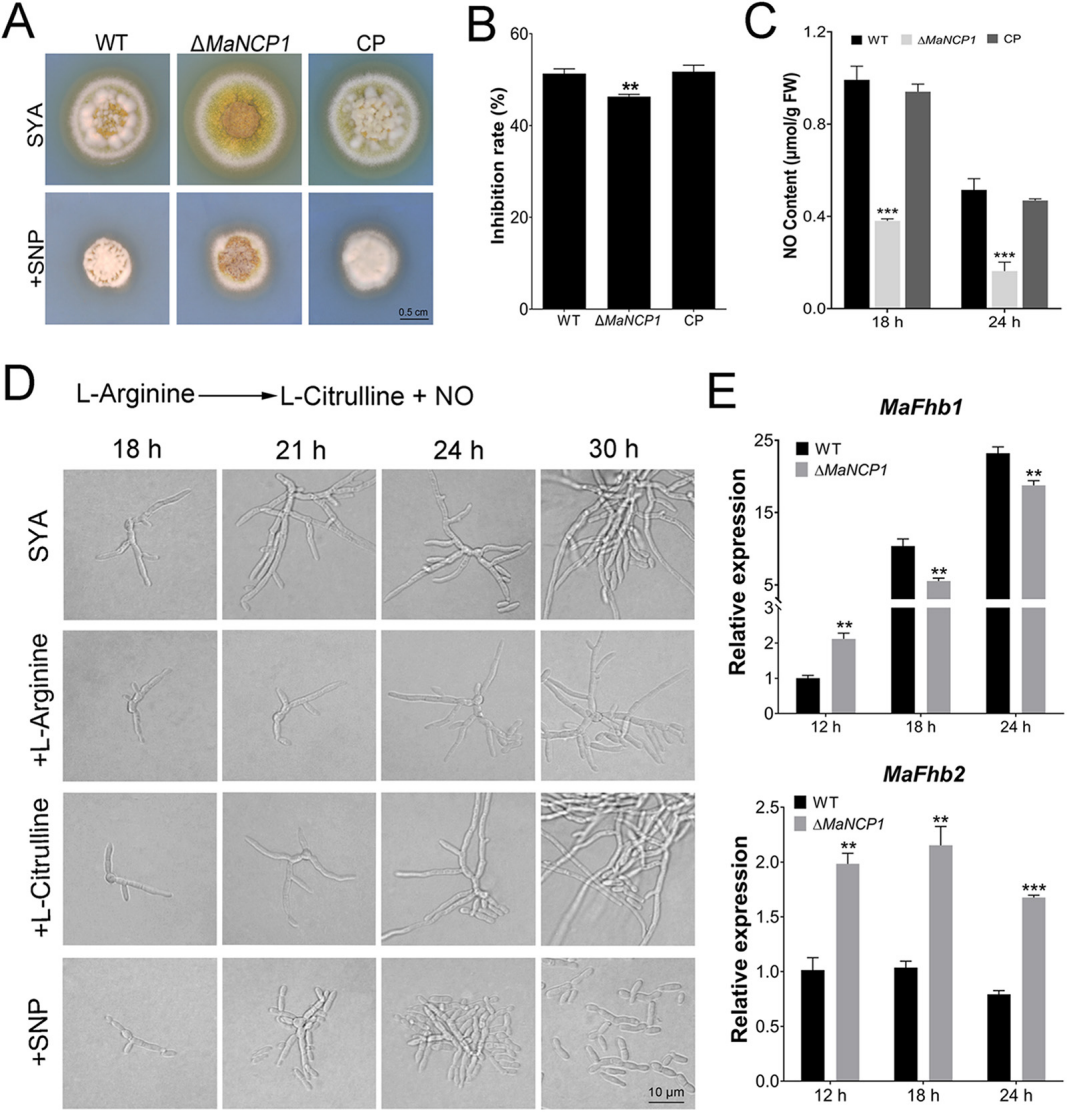
NO regulates the conidiation pattern shift in Δ*MaNCP1* strain. (A, B) Growth and inhibition rates of the WT, Δ*MaNCP1* and CP strains grown on SYA without or with SNP. (C) Determination of the NO content in the fungal strains on SYA medium over time. (D) Conidiation pattern of the Δ*MaNCP1* strain grown on SYA medium supplemented with 2 mM L-Arginine, 2 mM L-Citrulline or 3 mM SNP. A schematic diagram of the arginine metabolism pathway is presented above. (E) Relative expression of *MaFhb1* and *MaFhb2* in the WT and Δ*MaNCP1* strains cultured on SYA over time. n.s. no significant difference, *P > *0.05. *, *P < *0.05; **, *P < *0.01; ***, *P < *0.001.

**FIG 6:**
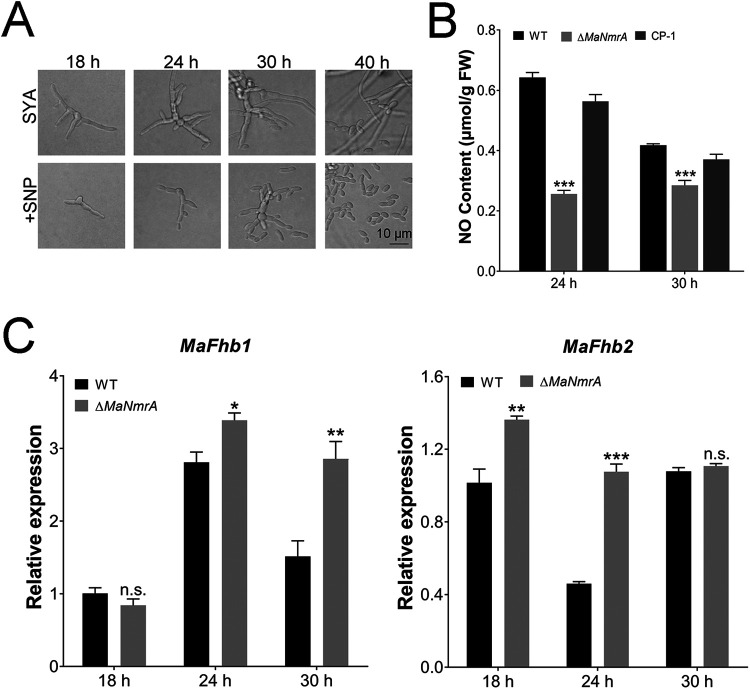
NO regulates the conidiation pattern shift in Δ*MaNmrA* strain. (A) Conidiation pattern of the Δ*MaNmrA* strain grown on SYA without or with 3 mM SNP. (B) Determination of the NO content in the WT, Δ*MaNmrA* and CP-1 strains on SYA medium over time. (C) Relative expression of *MaFhb1* and *MaFhb2* in the WT and Δ*MaNmrA* strains cultured on SYA over time. n.s. no significant difference, *P > *0.05. *, *P < *0.05; **, *P < *0.01; ***, *P < *0.001.

NO is an important signal molecule involved in the fungal adaptation to adverse environmental conditions (39), fungal virulence (33, 40–42), and conidial production (27, 32, 34, 35). Therefore, we tested whether the conidiation pattern shift regulated by MaNCP1 occurred with alterations to the NO content in M. acridum. The inhibition rate of the Δ*MaNCP1* strain was significantly lower than that of the WT or CP strain grown on SYA supplemented with the NO donor, sodium nitroprusside (SNP) (Fig. 5A and B), and the NO content of Δ*MaNCP1* strain was significantly lower than that of the WT or CP strain at 18 h and 24 h (Fig. 5C). To investigate the effect of the oxidative synthesis pathway of NO on the conidiation pattern regulated by *MaNCP1*, supplements L-arginine (the substrate), L-citrulline (a product) and SNP were added into SYA media, and it showed that only SNP supplements could restore microcycle conidiation in the Δ*MaNCP1* strain (Fig. 5D). The flavohemoglobin gene *FhbA* can promote the conversion of NO to nitrate in A. nidulans (43). Disruption of *MaNCP1* led to significant increases in the expression levels of two flavohemoglobin genes: *MaFhb1* (*MAC*_*06853*) at 12 h and *MaFhb2* (*MAC*_*09730*) at all tested time points (Fig. 5E). Similarly, the conidiation pattern of the Δ*MaNmrA* strain could be restored to microcycle conidiation when the Δ*MaNmrA* strain was grown on SYA medium with SNP (Fig. 6A), and the NO content was also significantly decreased in the Δ*MaNmrA* strain (Fig. 6B). The expression levels of *MaFhb1* and *MaFhb2* were significantly increased at the different time points in the Δ*MaNmrA* background (Fig. 6C).

### Overexpressing *MaNmrA* in the Δ*MaNCP1* strain can restore the shift to microcycle conidiation on SYA medium.

**FIG 7:**
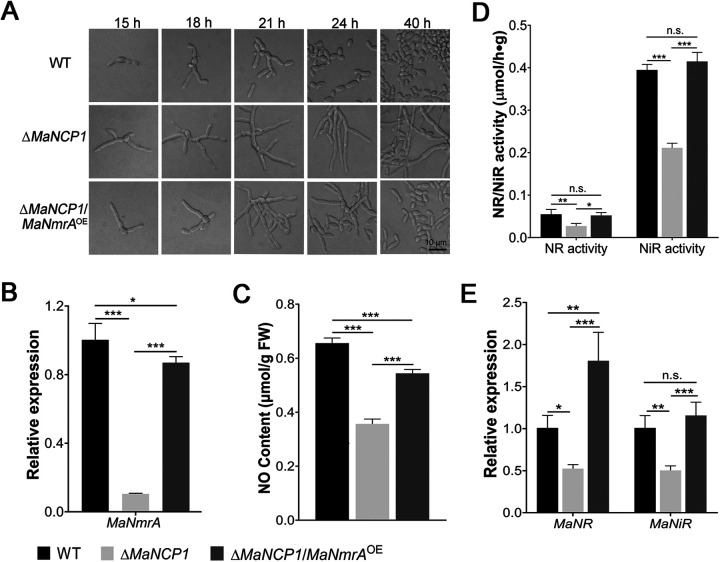
*MaNCP1* regulates the conidiation pattern shift by binding to *MaNmrA*. (A) Conidiation pattern of the Δ*MaNCP1*/*MaNmrA*^OE^ strain grown on SYA at 28°C over time. Δ*MaNCP1*/*MaNmrA*^OE^ refers to the strain overexpressing the *MaNmrA* gene in the Δ*MaNCP1* background. (B) Relative expression of *MaNmrA*. (C) Determination of the NO content in the fungal strains. (D) Determination of the NR and NiR activities in the fungal strains. (E) Relative expression of *MaNR* and *MaNiR*. Fungal samples were collected after 24 h of culture on SYA plates at 28°C. n.s. no significant difference, *P > *0.05. *, *P < *0.05; **, *P < *0.01; ***, *P < *0.001.

To further verify whether MaNCP1 regulates the conidiation pattern shift through its binding to *MaNmrA*, the *MaNmrA* gene was overexpressed in the Δ*MaNCP1* strain, Δ*MaNCP1/MaNmrA*^OE^ where the conidiation pattern was observed to be similar to that of the WT strain (Fig. 7A). The expression of *MaNmrA* in the Δ*MaNCP1*/*MaNmrA*^OE^ strain was significantly higher than that in the Δ*MaNCP1* strain (Fig. 7B). Moreover, the NO content in the Δ*MaNCP1*/*MaNmrA*^OE^ strain was significantly increased compared to the Δ*MaNCP1* strain (Fig. 7C). Furthermore, the activities of MaNR and MaNiR (Fig. 7D) and the expression levels of *MaNR* and *MaNiR* (Fig. 7E) were significantly increased in the Δ*MaNCP1*/*MaNmrA*^OE^ strain. Taken together, the data indicate that overexpressing *MaNmrA* in the Δ*MaNCP1* strain can promote nitrate assimilation to increase the intracellular NO content, leading to the restoration of microcycle conidiation.

**FIG 8:**
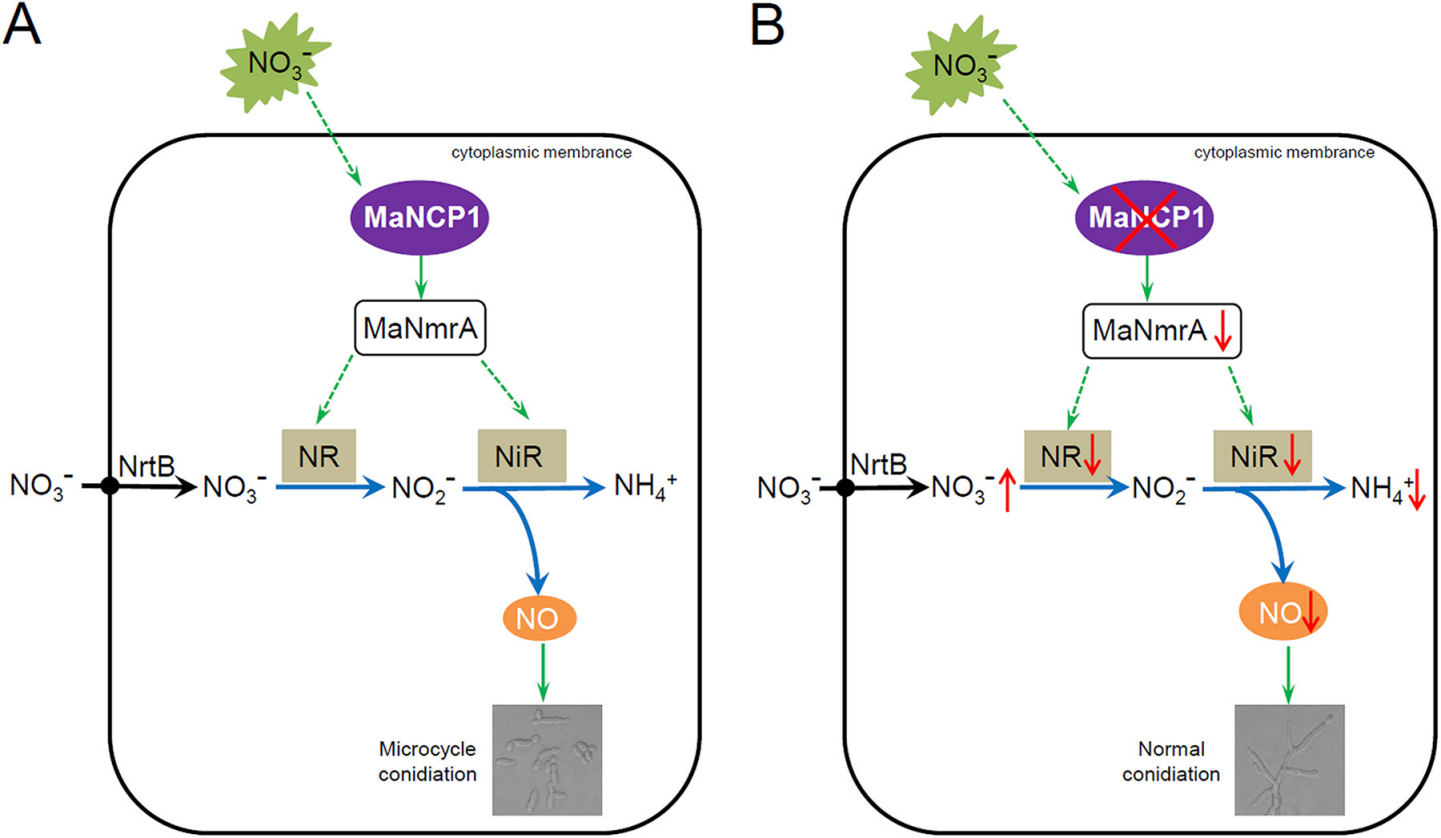
Schematic model of how MaNCP1 governs the conidiation pattern shift by regulating the nitrate metabolism in *M. acridum*. (A) MaNCP1 is involved in the nitrate assimilation pathway (the reductive pathway of NO synthesis) by binding to *MaNmrA* to promote the activities of key enzymes, such as nitrate reductase NR and nitrite reductase NiR, and the expression of their encoding genes to increase the intracellular NO content, leading to microcycle conidiation of *M. acridum* when grown on SYA medium. (B) Disruption of the *MaNCP1* gene significantly reduces the expression level of *MaNmrA* and impairs the nitrate assimilation to significantly decrease the intracellular NO content, resulting in the normal conidiation pattern in *M. acridum* when grown on SYA medium. The red fork indicates the deletion of the *MaNCP1* gene. The green arrows indicate positive regulation, blue arrows indicate biosynthesis and metabolic pathways. The red arrows indicate an increase or decrease in NR/NiR activity, the expression level of *MaNmrA* or the contents of nitrate, ammonium and NO in the absence of *MaNCP1.*

In summary, MaNCP1 is activated by nitrate and binds to *MaNmrA* to promote nitrate assimilation and the increase in intracellular NO content, leading to microcycle conidiation of *M. acridum* on SYA medium (Fig. 8A). In support, *MaNCP1* gene disruption leads to a significantly reduced expression of *MaNmrA*, with consequent impairment to nitrate assimilation and a significant decrease in NO content, resulting in the onset of normal conidiation of *M. acridum* on SYA medium (Fig. 8B).

## DISCUSSION

The conidial production of filamentous fungi is one of the most critical stages in their life cycle and plays important roles in the continued reproduction of the species and the maintenance of ecological diversity (2, 44). Here, we have studied the regulatory pathway governing the shift in conidiation pattern in response to nitrate in the model entomopathogenic fungus, M. acridum. MaNCP1, a protein with multiple C2H2 zinc finger domains, was found to mediate the reductive pathway of NO synthesis, namely, the nitrate assimilation pathway, to regulate the conidiation pattern in M. acridum. This study offers insights into novel technical means for improving the productivity of mycoinsecticides.

To date, homologs of MaNCP1 have not been reported in entomopathogenic fungi. The MaNCP1 protein contains similar domains to the mammalian Ikaros family members (Ikaros, Aiolos, Helios, Eos, and Pegasus), which all contain two C2H2 domain clusters: an N-terminal cluster composed of 3–4 zinc fingers and a C-terminal cluster containing 2 zinc fingers (45). In general, the N-terminal zinc fingers of the Ikaros family proteins are involved in binding to the DNA of target genes, while the C-terminal zinc fingers mediate protein-protein interactions (45, 46). Here, our results show that MaNCP1 has the typical characteristics of a transcription factor, and that the N-terminal of MaNCP1 protein is involved in the conidiation pattern shift. *MaNmrA*, a core gene in the NCR pathway, which also regulates the conidiation pattern shift of M. acridum, was identified in a screen for downstream target genes and was subsequently confirmed as a direct downstream target gene of MaNCP1. The NCR pathway is involved in regulating the expression of genes related to the assimilation and catabolism of multiple nitrogen sources (47). It has been reported that the core regulators in NCR pathway are not only involved in arginine metabolism (24), but also regulate the expression of nitrate reductase and nitrite reductase to promote nitrate assimilation (48) which, in fungi mainly involves the reductive pathway of NO synthesis.

The signaling molecule, NO, is involved in many fungal biological processes, such as in the adaptation of S. cerevisiae to adverse environmental conditions (39), the formation of appressoria in Blumeria graminis (40), and the development of conidia in N. crassa (28) and C. minitans (35). The reductive pathway of NO synthesis, namely, nitrate assimilation pathway, is a major pathway for producing NO in fungi, which has been confirmed in A. nidulans (27). In this study, we found that the transcription of the nitrate transporter encoding gene *MaNrtB* was significantly downregulated in Δ*MaNCP1* and Δ*MaNmrA* strains, suggesting a reduced import of nitrate. Nevertheless, the nitrate contents in the Δ*MaNCP1* and Δ*MaNmrA* strains were significantly higher than that in the WT or CP strains, indicating that the nitrate catabolism was significantly impaired in the absence of functional *MaNCP1* or *MaNmrA* genes in M. acridum. Consistently, the relative expression levels of *MaNR* and *MaNiR*, the activities of NR and NiR and the NO contents were significantly decreased in both the Δ*MaNCP1* and Δ*MaNmrA* strains. In addition, deletion of either *MaNCP1* or *MaNmrA* affected the ammonium assimilation, but the ammonium contents and the expression trends of key genes involved in ammonium assimilation were opposite, indicating that *MaNCP1* and *MaNmrA* played different roles in ammonium assimilation. It also indicated that the conidiation pattern shift regulated by *MaNCP1* and *MaNmrA* may be mainly mediated by the assimilation of nitrate, rather than ammonium. Furthermore, flavohemoglobin may be a further factor responsible for initiating changes in NO contents, since flavohemoglobin can catalyze the conversion of NO to produce nitrate and/or nitrite, thereby preventing nitrooxidative stress in cells caused by excessive NO accumulation (43, 49, 50). Here, we found that the flavohemoglobin gene, *MaFhb2*, was upregulated in the absence of *MaNCP1*, while both *MaFhb1* and *MaFhb2* were upregulated in the absence of *MaNmrA*.

Furthermore, studies have shown that the synthesis of NO in filamentous fungi can be independent of the NOS oxidative pathway (51), and an arginine-dependent biochemical route of NO synthesis, similar to that involving NOS, has recently been reported to be active in A. nidulans (52). However, we found that the conidiation pattern of Δ*MaNCP1* strain could not be restored when L-arginine or L-citrulline, the substrate and a product in the oxidative synthesis pathway of NO, were added to SYA medium, indicating that L-arginine metabolism plays a negligible role in regulating *MaNCP1-* mediated conidiation pattern shift. Nevertheless, we cannot rule out a role for an oxidative synthesis pathway in NO production during the conidiation pattern shift simply because no NOS homologues have not been identified in fungal genomes (41). Moreover, it was found that the overexpression of *MaNmrA* in the Δ*MaNCP1* strain could restore microcycle conidiation pattern and WT levels of NO content. This indicated that MaNCP1 regulated the conidiation pattern shift through affecting the reductive synthesis of NO by binding on *MaNmrA*.

Due to a lack of any obvious DNA binding sequence, NmrA must bind to some transcription factors to regulate the expression of downstream target genes. Under nitrogen sufficiency, NmrA can bind to the AreA protein, a core GATA transcription factor in the NCR pathway, to inhibit the expression of genes involved in nitrate assimilation (16, 19). Under nitrogen starvation, NIT-4, a homolog of the pathway-specific transcription factor, NirA, has also been confirmed as a downstream target gene of NmrA in N. crassa (53). In addition, the M. oryzae NmrA homologues can also interact with the other GATA transcription factors, PAS1 and ASD4 (22). Therefore, the targeted transcription factors interacting with NmrA might differ under different conditions. In this study, we have confirmed that the expression of *MaNmrA* was significantly downregulated in the absence of *MaNCP1* and the activity of key enzymes, NR and NiR, together with the expression of their encoding genes both decreased in the Δ*MaNCP1* and Δ*MaNmrA* strains that grown on SYA media. However, the culture conditions used in this study did not provide typical nitrogen repressed or de-repressive environments. In fact, the M. acridum WT strain on SYA medium did not exhibit the typical de-repression status in the NR and NiR activity assays (Fig. S9), implying that the selection of nitrogen source for use involved a more complex regulatory mechanism. Furthermore, the deletion of *MaAreA* did not affect the conidiation pattern of M. acridum on SYA medium. We therefore reasoned that *NmrA* plays a central role in regulating conidiation pattern shift on SYA medium, and that this functions independently of *AreA*. Thus, we postulate that NmrA may interact with alternative transcription factor(s) to activate nitrate catabolism during the conidiation pattern shift in M. acridum. Further research is now needed to identify the transcription factor(s) involved downstream of NmrA in the regulation of the conidiation pattern shift on SYA medium.

## MATERIALS AND METHODS

### Strains and culture conditions.

All mutants were generated from the wild-type *M. acridum* strain CQMa102 (WT) and grown on 1/4 SDAY media (10‰ glucose, 5‰ yeast extract, 2.5‰ peptone and 18‰ agar, wt/vol) or SYA media (5‰ yeast extract, 0.5‰ KCl, 1‰ KH_2_PO_4_, 30‰ sucrose, 0.5‰ MgSO_4_, 3‰ NaNO_3_, 0.01‰ MnSO_4_, 0.01‰ FeSO_4_ and 18‰ agar, wt/vol) at 28°C. The Y2HGold and Y187 yeast strains (Clontech, Palo Alto, CA, USA) were used in the autoactivation and yeast one-hybrid assays, respectively. Escherichia coli DH5α (Solarbio, Beijing, China) and Agrobacterium tumefaciens AGL1 (Solarbio, Beijing, China) were used for the recombinant plasmid manipulations and fungal transformations, respectively.

### Bioinformatics analyses.

All protein sequences used in this study were retrieved and downloaded from the NCBI genome database. cNLS mapper (https://nls-mapper.iab.keio.ac.jp/cgi-bin/NLS_Mapper_form.cgi) was used to predict the nuclear localization signal. Multiple sequence alignments were conducted with DNAMAN software (version 7). MEGA6.0 was used to construct the Neighbor-joining phylogenetic tree using the default settings with 1000 bootstrap replicates. The JASPAR 2020 database (http://jaspar.genereg.net/) (54) was used to analyze the putative binding sites of MaNCP1.

### Constructions of the mutants.

To construct the *MaNCP1*-disruption vectors pK2-SM-*MaNCP1*-F and pK2-SM-*MaNCP1*-R (Fig. S2A), about 1,200-bp up- and downstream fragments of *MaNCP1* were amplified from the genomic DNA of WT strain, followed by ligating into pK2-SM-F and pK2-SM-R vectors as described previously (55). The *MaAreB*- and *MaNmrA*-disruption strains were constructed previously (37, 38). The constructions of the disruption vectors of *MAC_02692*, *MAC_03846*, *MAC_02196* and *MaAreA* were made similar to that of *MaNCP1*. To construct the *MaNCP1* complementary vector pK2-*MaNCP1*-sur (Fig. S2B), a 2,482-bp DNA sequence of *MaNCP1* with its adjacent 1,200-bp upstream fragment and a 1,200-bp downstream fragment were amplified to insert into pK2-Sur vector (56). To further explore the functions of the two clusters of zinc finger domains in MaNCP1, fusion PCR was used to amplify the C2H2 zinc fingers domain deletion fragments (the details were addressed in Table S2 and S3), which were ligated into pK2-SM-F, and about a 1.3 kb fragment at the 3′ flanking of *MaNCP1* gene was amplified and inserted into the PK2-SM-R vector. These recombinant plasmids were used to screen the C2H2 zinc fingers domain deletion mutants (Fig. S2D). The N- and C-terminal zinc finger cluster mutants were labeled as MaNCP1*^ΔN^* and MaNCP1*^ΔC^*, respectively. The combined N- and C-terminal zinc finger cluster deletion mutant was marked as MaNCP1*^ΔN+C^*. To determine the subcellular localization of MaNCP1, the overexpression vector pK2-*pgpdA*-MaNCP1-eGFP-sur was constructed. A 2,479-bp DNA sequence of MaNCP1 was amplified with primers MaNCP1-OF/MaNCP1-OR and inserted into pK2-*pgpdA-*eGFP-sur vector, which carried a constitutive promoter *pgpdA* and an enhanced green fluorescent protein (eGFP). To overexpress the *MaNmrA* gene in the Δ*MaNCP1* strain, a 1,383-bp DNA fragment of *MaNmrA* and *pgpdA* promoter were cloned and inserted into pK2-Nat (57), which harbors a *Nat* cassette, to form the *MaNmrA* overexpression vector pK2-*pgpdA*-*MaNmrA*-Nat. The recombinant plasmids were transferred into AGL1 for fungal transformation. Knockout transformants (e.g., Δ*MaNCP1*) were selected on Czapek-dox agar (CZA) media with 500 μg/mL glufosinate-ammonium (Sigma, St. Louis, MO, USA). The complementary (CP) and overexpression transformants were screened on CZA media with 20 μg/mL chlorimuron ethyl (Sigma, Bellefonte, PA, USA) or 75 μg/mL nourseothricin sulfate (Harveybio, Beijing, China). Transformants were identified by PCR and Southern blotting with DIG High Prime DNA Labeling and Detection Starter Kit I (Roche, Basel, Switzerland) (Fig. S2C and E). Primers used in this study are listed in Table S3.

### Microscopic observation of the conidiation pattern.

The fresh and mature conidia (15-day-old) of fungal strains grown on 1/4SDAY media were collected to prepare the conidial suspensions (10^7^ conidia/mL). An aliquot of 100 μL conidial suspension of each fungal strain was spread evenly onto SYA plate and cultured at 28°C for several hours, followed by cutting about 1 cm^2^ media contained the fungal cultures to observe the fungal growth. Images were taken with a digital light microscope (MOTIC, Xiamen, China).

### Analyses of hyphal polar growth and subcellular localization.

To determine the number and the length of hyphal cells, the hyphae of the WT, Δ*MaNCP1* and CP strains grown on SYA plates were stained with calcofluor white (CFW) after 14 h of culture, followed by incubating at room temperature for 20–30 min and photographing with a fluorescence microscope (Nikon Y-TV55, Tokyo, Japan). To observe the hyphal polar growth, hyphae were stained with FM4-64 (Invitrogen, Waltham, MA, USA) as described previously (58). For the subcellular localization of MaNCP1, the *MaNCP1::eGFP*-overexpression strain was cultured on SYA and visualized through fluorescence microscopy.

### Transcriptional activity assay.

A 2,271-bp cDNA fragment encoding MaNCP1 was cloned with the primer pair of MaNCP1-F/MaNCP1-R (Table S3 in the online supplemental material) and ligated into the yeast expression vector pGBKT7 to form the recombinant plasmid pGBKT7-MaNCP1, followed by transforming into the yeast strain Y2HGold. The positive transformants were spread on an SD/-Trp solid plate containing 25 μg/mL X-α-Gal and 125 ng/mL Aureobasidin A (AbA) (i.e., SD/-Trp/X-α-Gal/AbA) to determine whether MaNCP1 has autoactivation activity, according to the Yeastmaker Gold Yeast Two-Hybrid System User Manual (Clontech).

### Yeast one-hybrid assay.

The cDNA encoding MaNCP1 was amplified and ligated into pGADT7 vector to construct the recombinant plasmid pGADT7-MaNCP1. The promoter sequence of *MaNmrA* was amplified and ligated into the pHIS2 vector to generate pHIS2-*MaNmrA*, followed by transferring into the Y187 strain to construct Y187 (pHIS2-*MaNmrA*) and spreading on the selective media lacking tryptophan and histidine (SD/-Trp/-His) with different concentration of 3-Amino-1,2,4-triazole (3-AT), a competitive inhibitor of HIS3 expression products, to suppress the leakage expression of HIS3 products at the background level and eliminate false positives. Then, pGADT7-MaNCP1 and pHIS2-*MaNmrA* were co-transformed into the Y187 and spotted on the selective medium lacking leucine, tryptophan and histidine (SD/-Leu/-Trp/-His) with or without 3-AT to observe the growth of the yeast. The preparation and transformation of yeast competent cells were conducted according to the Yeastmaker Yeast Transformation System2 User Manual (Clontech). Y187 cells transformed with pGADT7-53 and pHIS2-53 vectors was used as the positive control. Y187 cells transformed with empty pHIS2 and pGADT7-MaNCP1 or empty pGADT7 and pHIS2-*MaNmrA* vectors were used as the negative control.

### EMSA analysis.

The cDNA sequence of the zinc finger cluster at the N-terminal of MaNCP1 was subcloned and ligated into the expression vector pCold-MBP-TEV to construct the fusion recombinant plasmid pCold-MaNCP1-N. The expressed protein MaNCP1-N was purified by ÄKTA *prime* plus protein purification system (GE Healthcare, Stockholm, Sweden), followed by concentration and de-salting in a 30-kDa cutoff ultrafiltration tube (Millipore Amicon). The promoter region of *MaNmrA*, containing a putative binding site for MnNCP1 was amplified with primers Probe-F/Probe-R (Table S3) to produce a 110 bp probe which, was labeled with biotin via the EMSA Probe Biotin Labeling Kit (Beyotime, Shanghai, China) (59). The Chemiluminescent EMSA kit (Beyotime, Shanghai, China) was used for the EMSA (59). The unlabeled probe was added in a 100-fold excess.

### qRT-PCR assay.

Total RNAs were isolated from conidia and/or hyphae of the WT or Δ*MaNCP1* strain. To further confirm the expression patterns of *MAC_00186*, *MAC_08242*, *MAC_02692*, *MAC_03472*, *MAC_03846*, *MAC_03700*, *MAC_04326* and *MAC_06473* in the transcriptome data previously (15), samples of the WT strain were collected after growing on the 1/4SDAY, SYA, SYA+Nitrate (SYA+N), SYA+Sucrose (SYA+C) or SYA+Phosphate (SYA+P) plates at 28°C for 21 h. To analyze the expression of *MaAreA*, *MaAreB*, *MaNmrA*, *MaNrtB*, *MaNR*, *MaNiR*, *MaFhb1*, *MaFhb2*, etc., fungal samples were harvested from SYA at different time points between 0 and 24 h. Total RNA extraction and cDNA synthesis were performed as per the manufacturer’s instructions using Fungal RNA Kit (OMEGA, USA) and PrimeScript RT reagent kit with genomic DNA Eraser (TaKaRa, Dalian, China), respectively. SYBR Premix Ex Taq (TaKaRa, Dalian, China) was used for qRT-PCR. The relative expression level was quantified using the 2^-ΔΔCt^ method with *gpdh* gene (*MAC_09584*) as the internal control gene. Each treatment was performed with three biological replicates. The primers used are listed in Table S3.

### Nitrate and nitrite reductase activity assays and detection of NO, nitrate and ammonium content.

Fungal samples grown on SYA for 24 h and/or 30 h at 28°C were collected and ground into powder in liquid nitrogen. To assay the nitrate reductase (NR) activity, the nitrite reductase (NiR) activity and the ammonium content, the lysis buffer were added into the powdered samples to the mass/volume ratio specified in the operation manual of the respective detection kits (Comin, Suzhou, China). The suspensions were then centrifuged at 10,000 × *g* and 4°C for 15 min, and the supernatant collected for the subsequent biochemical measurements as per the manufacturer’s instructions. To assay the nitrate content, 0.1 g fresh sample was homogenized in 1 mL ddH_2_O at room temperature, followed by shaking at 90°C for 30 min. After cooling to room temperature, the supernatant were centrifuged at 10,000 × *g* for 20 min to be measured as described in the manufacturer’s manual (Comin, Suzhou, China). Fungal cultures on SYA at 28°C for hours were collected and washed three times with sterile water for NO content detection using a visible spectrophotometric NO content detection kit (Solarbio, Beijing, China) as described previously (60).

### Transcriptomic analysis.

To reveal the mechanism underlying the MaNCP1 regulation of the conidiation pattern shift, RNA-seq was performed to identify the differentially expressed genes (DEGs) in WT *versus* Δ*MaNCP1*. Samples of the WT and Δ*MaNCP1* strains after 18 h of culture on SYA were collected for RNA extraction. Approximately 10 μg DNA-free RNA from each fungal sample was submitted to BGISEQ-500 (BGI, Shenzhen, China) in the Beijing Genomics Institution (Wuhan, China) with three biological replicates for each fungal strain. The DEGs were defined as those displaying a log_2_ Δ*MaNCP1*/WT ratios ≥ 1 or ≤ −1 with a false-discover rate (FDR) ≤ 0.001. The DEGs were annotated according to the NCBI protein databases.

### Statistical analysis.

The data, shown in mean ± SE, were analyzed by ANOVA (one-way analysis of variance) using SPSS 24.0 program (SPSS Inc, Chicago, IL, USA).

### Data availability.

RNA-seq data had been uploaded to the NCBI BioProject database under the accession number PRJNA748190.

## References

[1] Wang C, Wang S. 2017. Insect pathogenic fungi: genomics, molecular interactions, and genetic improvements. Annu Rev Entomol 62:73–90. doi:10.1146/annurev-ento-031616-035509. 27860524

[2] Hawker LE, Madelin MF. 1976. The dormant spore, p 1–72. *In* Webber DJ, Hess WM (ed), The fungal spore. Wiley, New York.

[3] Wolken WAM, Tramper J, van der Werf MJ. 2002. Toxicity of terpenes to spores and mycelium of P*enicillium digitatum*. Biotechnol Bioeng 80:685–690. doi:10.1002/bit.10435. 12378610

[4] Tong X, Wang Y, Yang P, Wang C, Kang L. 2020. Tryptamine accumulation caused by deletion of *MrMao-1* in *Metarhizium* genome significantly enhances insecticidal virulence. PLoS Genet 16:e1008675. doi:10.1371/journal.pgen.1008675. 32271756 PMC7173932

[5] Mahato SB, Majumdar I. 1993. Current trends in microbial steroid biotransformation. Phytochemistry 34:883–898. doi:10.1016/s0031-9422(00)90685-x. 7764240

[6] Howell CR, Hanson LE, Stipanovic RD, Puckhaber LS. 2000. Induction of terpenoid syntheis in cotton roots and control of *Rhizoctonia solani* by seed treatment with *Trichoderma virens*. Phytopathology 90:248–252. doi:10.1094/PHYTO.2000.90.3.248. 18944616

[7] Wolken WAM, van der Werf MJ. 2001. Geraniol biotransformation-pathway in spores of *Penicillium digitatum*. Appl Microbiol Biotechnol 57:731–737. doi:10.1007/s002530100821. 11778886

[8] Jung B, Kim S, Lee J. 2014. Microcyle conidiation in filamentous fungi. Mycobiology 42:1–5. doi:10.5941/MYCO.2014.42.1.1. 24808726 PMC4004940

[9] Zhang S, Peng G, Xia Y. 2010. Microcycle conidiation and the conidial properties in the entomopathogenic fungus *Metarhizium acridum* on agar medium. Biocontrol Sci Techn 20:809–819. doi:10.1080/09583157.2010.482201.

[10] Anderson JG, Smith JE. 1971. The production of conidiophores and conidia by newly germinated conidia of *Aspergillus niger* (microcycle conidiation). J Gen Microbiol 69:185–197. doi:10.1099/00221287-69-2-185. 5146836

[11] Pažout J, Schröder P. 1988. Microcycle conidiation in submerged cultures of *Penicillium cyclopium* attained without temperature changes. J Gen Microbiol 134:2685–2692. doi:10.1099/00221287-134-10-2685. 2908204

[12] Vezina C, Singh K, Sehgal SN. 1965. Sporulation of filamentous fungi in submerged culture. Mycologia 57:722–736. doi:10.2307/3757008.

[13] Sekiguchi J, Gaucher GM, Costerton JW. 1975. Microcycle conidiation in *Penicillium urticae*: an ultrastructural investigation of conidiogenesis. Can J Microbiol 21:2069–2083. doi:10.1139/m75-296. 1220869

[14] Slade SJ, Harris RF, Smith CS, Andrews JH. 1987. Microcycle conidiation and spore-carrying capacity of *Colletotrichum gloeosporioides* on solid media. Appl Environ Microbiol 53:2106–2110. doi:10.1128/aem.53.9.2106-2110.1987. 16347433 PMC204065

[15] Wang ZL, Jin K, Xia YX. 2016. Transcriptional analysis of the conidiation pattern shift of the entomopathogenic fungus *Metarhizium acridum* in response to different nutrients. BMC Genomics 17:586. doi:10.1186/s12864-016-2971-0. 27506833 PMC4979188

[16] Marzluf GA. 1997. Genetic regulation of nitrogen metabolism in the fungi. Microbiol Mol Biol Rev 61:17–32. doi:10.1128/mmbr.61.1.17-32.1997. 9106362 PMC232598

[17] Ravagnani A, Gorfinkiel L, Langdon T, Diallinas G, Adjadj E, Demais S, Gorton D, Arst HN, Scazzocchio C. 1997. Subtle hydrophobic interactions between the seventh residue of the zinc finger loop and the first base of an HGATAR sequence determine promoter-specific recognition by the *Aspergillus nidulans* GATA factor AreA. EMBO J 16:3974–3986. doi:10.1093/emboj/16.13.3974. 9233807 PMC1170021

[18] Pan H, Feng B, Marzluf GA. 1997. Two distinct protein-protein interactions between the NIT2 and NMR regulatory proteins are required to establish nitrogen metabolite repression in *Neurospora crassa*. Mol Microbiol 26:721–729. doi:10.1046/j.1365-2958.1997.6041979.x. 9427402

[19] Wong KH, Hynes MJ, Todd RB, Davis MA. 2007. Transcriptional control of *nmrA* by the bZIP transcription factor MeaB reveals a new level of nitrogen regulation in *Aspergillus nidulans*. Mol Microbiol 66:534–551. doi:10.1111/j.1365-2958.2007.05940.x. 17854403

[20] Kotaka M, Johnson C, Lamb HK, Hawkins AR, Ren J, Stammers DK. 2008. Structural analysis of the recognition of the negative regulator NmrA and DNA by the zinc finger from the GATA-type transcription factor AreA. J Mol Biol 381:373–382. doi:10.1016/j.jmb.2008.05.077. 18602114

[21] Polley SD, Caddick MX. 1996. Molecular characterisation of meaB, a novel gene affecting nitrogen metabolite repression in *Aspergillus nidulans*. FEBS Lett 388:200–205. doi:10.1016/0014-5793(96)00541-8. 8690087

[22] Wilson RA, Gibson RP, Quispe CF, Littlechild JA, Talbot NJ. 2010. An NADPH-dependent genetic switch regulates plant infection by the rice blast fungus. Proc Natl Acad Sci USA 107:21902–21907. doi:10.1073/pnas.1006839107. 21115813 PMC3003025

[23] Fernandez J, Wright JD, Hartline D, Quispe CF, Madayiputhiya N, Wilson RA. 2012. Principles of carbon catabolite repression in the rice blast fungus: Tps1, Nmr1-3, and a MATE–family pump regulate glucose metabolism during infection. PLoS Genet 8:e1002673. doi:10.1371/journal.pgen.1002673. 22570632 PMC3342947

[24] Macios M, Caddick MX, Weglenski P, Scazzocchio C, Dzikowska A. 2012. The GATA factors AREA and AREB together with the co-repressor NMRA, negatively regulate arginine catabolism in *Aspergillus nidulans* in response to nitrogen and carbon source. Fungal Genet Biol 49:189–198. doi:10.1016/j.fgb.2012.01.004. 22300944

[25] Han X, Qiu M, Wang B, Yin WB, Nie X, Qin Q, Ren S, Yang K, Zhang F, Zhuang Z, Wang S. 2016. Functional Analysis of the Nitrogen Metabolite Repression Regulator Gene *nmrA* in *Aspergillus flavus*. Front Microbiol 7:1794. 27933036 10.3389/fmicb.2016.01794 PMC5122588

[26] Zhao Y, Lim J, Xu J, Yu JH, Zheng W. 2020. Nitric oxide as a developmental and metabolic signal in filamentous fungi. Mol Microbiol 113:872–882. doi:10.1111/mmi.14465. 31968137

[27] Marcos AT, Ramos MS, Marcos JF, Carmona L, Strauss J, Cánovas D. 2016. Nitric oxide synthesis by nitrate reductase is regulated during development in *Aspergillus*. Mol Microbiol 99:15–33. doi:10.1111/mmi.13211. 26353949 PMC4982101

[28] Ninnemann H, Maier J. 1996. Indications for the occurrence of nitric oxide synthases in fungi and plants and the involvement in photoconidiation of *Neurospora crassa*. Photochem Photobiol 64:393–398. doi:10.1111/j.1751-1097.1996.tb02477.x. 8760579

[29] Maier J, Hecker R, Rockel P, Ninnemann H. 2001. Role of nitric oxide synthase in the light-induced development of sporangiophores in *Phycomyces blakesleeanus*. Plant Physiol 126:1323–1330. doi:10.1104/pp.126.3.1323. 11457983 PMC116489

[30] Wang J, Higgins VJ. 2005. Nitric oxide has a regulatory effect in the germination of conidia of *Colletotrichum coccodes*. Fungal Genet Biol 42:284–292. doi:10.1016/j.fgb.2004.12.006. 15749048

[31] Vieira ALG, Linares E, Augusto O, Gomes SL. 2009. Evidence of a Ca^2+^-NO-cGMP signaling pathway controlling zoospore biogenesis in the aquatic fungus *Blastocladiella emersonii*. Fungal Genet Biol 46:575–584. doi:10.1016/j.fgb.2009.04.002. 19393757

[32] Li B, Fu YP, Jiang DH, Xie JT, Cheng JS, Li GQ, Hamid MI, Yi XH. 2010. Cyclic GMP as a second messenger in the nitric oxide-mediated conidiation of the mycoparasite *Coniothyrium minitans*. Appl Environ Microbiol 76:2830–2836. doi:10.1128/AEM.02214-09. 20208018 PMC2863460

[33] Ding Y, Gardiner DM, Xiao D, Kazan K. 2020. Regulators of nitric oxide signaling triggered by host perception in a plant pathogen. Proc Natl Acad Sci USA 117:11147–11157. doi:10.1073/pnas.1918977117. 32376629 PMC7245131

[34] Marcos AT, Ramos MS, Schinko T, Strauss J, Canovas D. 2020. Nitric oxide homeostasis is required for light-dependent regulation of conidiation in *Aspergillus*. Fungal Genet Biol 137:103337. doi:10.1016/j.fgb.2020.103337. 31991229

[35] Gong X, Fu Y, Jiang D, Li G, Yi X, Peng Y. 2007. L-Arginine is essential for conidiation in the filamentous fungus *Coniothyrium minitans*. Fungal Genet Biol 44:368–1379. 17897846 10.1016/j.fgb.2007.07.007

[36] Cao H, Huang P, Zhang L, Shi Y, Sun D, Yan Y, Liu X, Dong B, Chen G, Snyder JH, Lin F, Lu J. 2016. Characterization of 47 Cys2-His2 zinc finger proteins required for the development and pathogenicity of the rice blast fungus *Magnaporthe oryzae*. New Phytol 211:1035–1051. doi:10.1111/nph.13948. 27041000

[37] Li CC, Zhang QP, Xia YX, Jin K. 2021. MaAreB, a GATA transcription factor, is involved in nitrogen source utilization, stress tolerances and virulence in *Metarhizium acridum*. JoF 7:512. doi:10.3390/jof7070512. 34198996 PMC8305397

[38] Li CC, Zhang QP, Xia YX, Jin K. 2021. MaNmrA, a negative transcription regulator in nitrogen catabolite repression pathway, contributes to nutrient utilization, stress resistance and virulence in entomopathogenic fungus *Metarhizium acridum*. Biology 10:1167. doi:10.3390/biology10111167. 34827160 PMC8615229

[39] Domitrovic T, Palhano FL, Barja-Fidalgo C, DeFreitas M, Orlando MTD, Fernandes PMB. 2003. Role of nitric oxide in the response of *Saccharomyces cerevisiae* cells to heat shock and high hydrostatic pressure. FEMS Yeast Res 3:341–346. doi:10.1016/S1567-1356(03)00039-4. 12748047

[40] Prats E, Carver TLW, Mur LAJ. 2008. Pathogen-derived nitric oxide influences formation of the appressorium infection structure in the phytopathogenic fungus *Blumeria graminis*. Res Microbiol 159:476–480. doi:10.1016/j.resmic.2008.04.001. 18554873

[41] Samalova M, Johnson J, Illes M, Kelly S, Fricker M, Gurr S. 2013. Nitric oxide generated by the rice blast fungus *Magnaporthe oryzae* drives plant infection. New Phytol 197:207–222. doi:10.1111/j.1469-8137.2012.04368.x. 23072575

[42] Hromatka BS, Noble SM, Johnson AD. 2005. Transcriptional response of *Candida albicans* to nitric oxide and the role of the *YHB1* gene in nitrosative stress and virulence. Mol Biol Cell 16:4814–4826. doi:10.1091/mbc.e05-05-0435. 16030247 PMC1237085

[43] Schinko T, Berger H, Lee W, Gallmetzer A, Pirker K, Pachlinger R, Buchner I, Reichenauer T, Güldener U, Strauss J. 2010. Transcriptome analysis of nitrate assimilation in *Aspergillus nidulans* reveals connections to nitric oxide metabolism. Mol Microbiol 78:720–738. doi:10.1111/j.1365-2958.2010.07363.x. 20969648 PMC3020322

[44] Krijgsheld P, Bleichrodt R, van Veluw GJ, Wang F, Müller WH, Dijksterhuis J, Wösten HAB. 2013. Development in *Aspergillus*. Stud Mycol 74:1–29. doi:10.3114/sim0006. 23450714 PMC3563288

[45] Perdomo J, Holmes M, Chong B, Crossley M. 2000. Eos and pegasus, two members of the Ikaros family of proteins with distinct DNA binding activities. J Biol Chem 275:38347–38354. doi:10.1074/jbc.M005457200. 10978333

[46] Morgan B, Sun L, Avitahl N, Andrikopoulos K, Ikeda T, Gonzales E, Wu P, Neben S, Georgopoulos K. 1997. Aiolos, a lymphoid restricted transcription factor that interacts with Ikaros to regulate lymphocyte differentiation. EMBO J 16:2004–2013. doi:10.1093/emboj/16.8.2004. 9155026 PMC1169803

[47] Arst HN, Jr, Cove DJ. 1973. Nitrogen metabolite repression in *Aspergillus nidulans*. Mol Gen Genet 126:111–141. doi:10.1007/BF00330988. 4591376

[48] Narendja F, Goller SP, Wolschek M, Strauss J. 2002. Nitrate and the GATA factor AreA are necessary for in vivo binding of NirA, the pathway-specific transcriptional activator of *Aspergillus nidulans*. Mol Microbiol 44:573–583. doi:10.1046/j.1365-2958.2002.02911.x. 11972792

[49] Liu L, Zeng M, Hausladen A, Heitman J, Stamler JS. 2000. Protection from nitrosative stress by yeast flavohemoglobin. Proc Natl Acad Sci USA 97:4672–4676. doi:10.1073/pnas.090083597. 10758168 PMC18291

[50] de Jesús-Berrios M, Liu L, Nussbaum JC, Cox GM, Stamler JS, Heitman J. 2003. Enzymes that counteract nitrosative stress promote fungal virulence. Curr Biol 13:1963–1968. doi:10.1016/j.cub.2003.10.029. 14614821

[51] Pengkit A, Jeon SS, Son SJ, Shin JH, Baik KY, Choi EH, Park G. 2016. Identification and functional analysis of endogenous nitric oxide in a filamentous fungus. Sci Rep 6:30037. doi:10.1038/srep30037. 27425220 PMC4948021

[52] Franco-Cano A, Marcos AT, Strauss J, Cánovas D. 2021. Evidence for an arginine-dependent route for the synthesis of NO in the model filamentous fungus *Aspergillus nidulans*. Environ Microbiol 23:6924–6939. doi:10.1111/1462-2920.15733. 34448331

[53] Huberman LB, Wu VW, Kowbel DJ, Lee J, Daum C, Grigoriev IV, O’Malley RC, Glass NL. 2021. DNA affinity purification sequencing and transcriptional profiling reveal new aspects of nitrogen regulation in a filamentous fungus. Proc Natl Acad Sci USA 118:e2009501118. doi:10.1073/pnas.2009501118. 33753477 PMC8020665

[54] Fornes O, Castro-Mondragon JA, Khan A, van der Lee R, Zhang X, Richmond PA, Modi BP, Correard S, Gheorghe M, Baranasic D, Santana-Garcia W, Tan G, Cheneby J, Ballester B, Parcy F, Sandelin A, Lenhard B, Wasserman WW, Mathelier A. 2020. JASPAR 2020: update of the open-access database of transcription factor binding profiles. Nucleic Acids Res 48:D87–D92. doi:10.1093/nar/gkz1001. 31701148 PMC7145627

[55] Du Y, Jin K, Xia Y. 2018. Involvement of *MaSom1*, a downstream transcriptional factor of cAMP/PKA pathway, in conidial yield, stress tolerances, and virulence in *Metarhizium acridum*. Appl Microbiol Biotechnol 102:5611–5623. doi:10.1007/s00253-018-9020-7. 29713793

[56] Jin K, Ming Y, Xia YX. 2012. *MaHog1*, a Hog1-type mitogen-activated protein kinase gene, contributes to stress tolerance and virulence of the entomopathogenic fungus *Metarhizium acridum*. Microbiology (Reading) 158:2987–2996. doi:10.1099/mic.0.059469-0. 23038805

[57] Zhao T, Wen Z, Xia Y, Jin K. 2020. The transmembrane protein MaSho1 negatively regulates conidial yield by shifting the conidiation pattern in *Metarhizium acridum*. Appl Microbiol Biotechnol 104:4005–4015. doi:10.1007/s00253-020-10523-0. 32170386

[58] Araujo-Palomares CL, Riquelme M, Castro-Longoria E. 2009. The polarisome component SPA-2 localizes at the apex of *Neurospora crassa* and partially colocalizes with the Spitzenkörper. Fungal Genet Biol 46:551–563. doi:10.1016/j.fgb.2009.02.009. 19281855

[59] Wang Z, Tian X, Zhao Q, Liu Z, Li X, Ren Y, Tang J, Fang J, Xu Q, Bu Q. 2018. The E3 ligase drought hypersensitive negatively regulates cuticular wax biosynthesis by promoting the degradation of transcription factor ROC4 in rice. Plant Cell 30:228–244. doi:10.1105/tpc.17.00823. 29237723 PMC5810576

[60] Peng X, Zhu L, Guo J, Sun Z, Zhao M, Zhan X. 2020. Enhancing biocompatibility and neuronal anti-inflammatory activity of polymyxin B through conjugation with gellan gum. Int J Biol Macromol 147:734–740. doi:10.1016/j.ijbiomac.2019.12.200. 31883895

