# Supplementary material for: MaNCP1, a C2H2 Zinc Finger Protein, Governs the Conidiation Pattern Shift through Regulating the Reductive Pathway for Nitric Oxide Synthesis in the Filamentous Fungus Metarhizium acridum
Source: Microbiol Spectr. 2022 May 10;10(3):e00538-22. doi: 10.1128/spectrum.00538-22 (PMC9241723; doi:10.1128/spectrum.00538-22)
Supplement: SUPPLEMENTAL FILE 1 — Supplemental material. Download spectrum.00538-22-s001.pdf, PDF file, 2.5 MB [file spectrum.00538-22-s001.pdf]

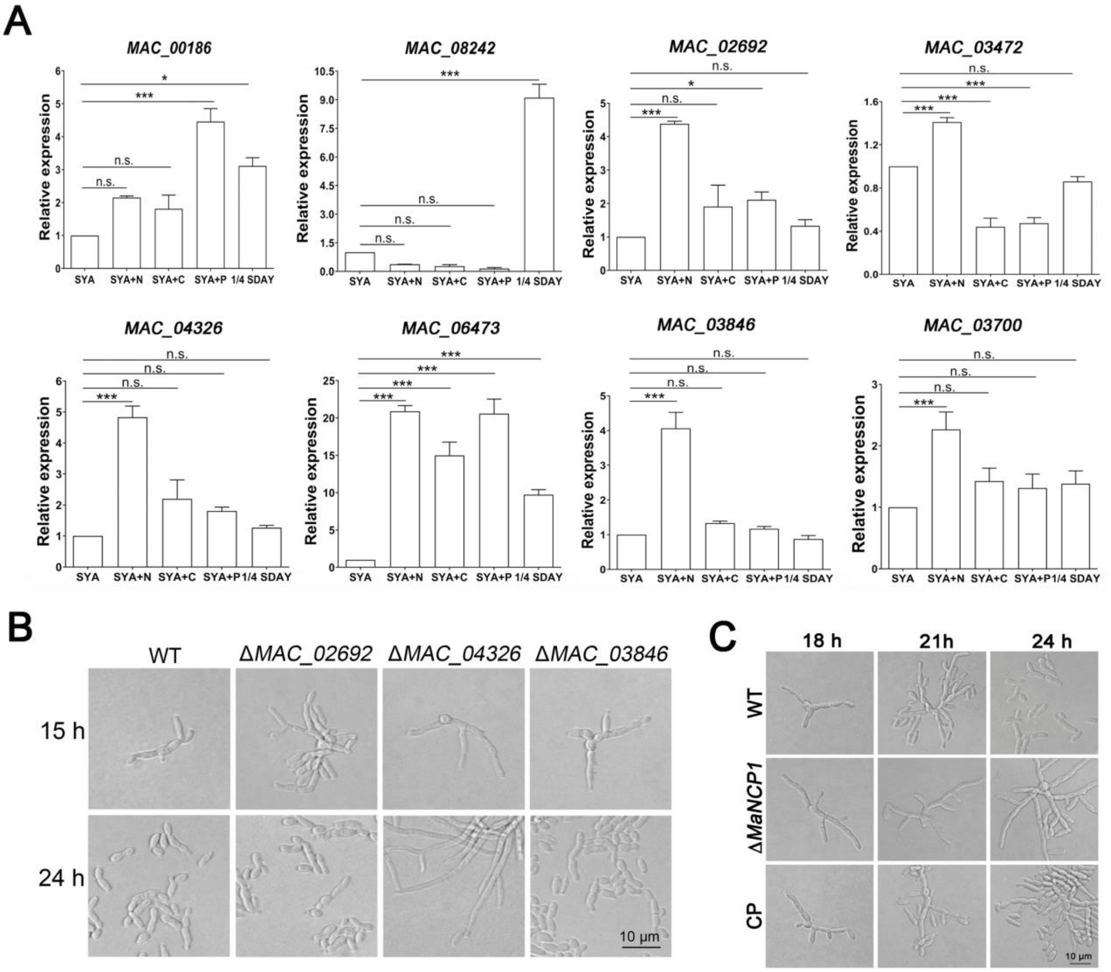

Fig. S1 Verification of transcriptome data. (**A**) Verification of the relative expression of the selected genes by qRT-PCR. The genes, *MAC_00186*, *MAC_08242*, *MAC_02692*, *MAC_03472*, *MAC_03846*, *MAC_03700*, *MAC_04326* and *MAC_06473*, were screened from the differential expression library of $NaNO_3$. Samples of WT strain were respectively cultured on 1/4SDAY, SYA, SYA+Nitrate (SYA+N), SYA+Sucrose (SYA+C) or SYA+Phosphate (SYA+P) media for 21 h, as described previously (15). n.s. no significant difference, $p > 0.05$. *$p < 0.05$, **$p < 0.01$, ***$p < 0.001$. (**B**) Conidiation pattern of the mutants grown on SYA media at 28℃ for hours. (**C**) Conidiation pattern of the WT, Δ*MaNCP1* and CP strains grown on SYA. WT, wild-type. Δ, the deletion transformants. CP, the complementary strain.

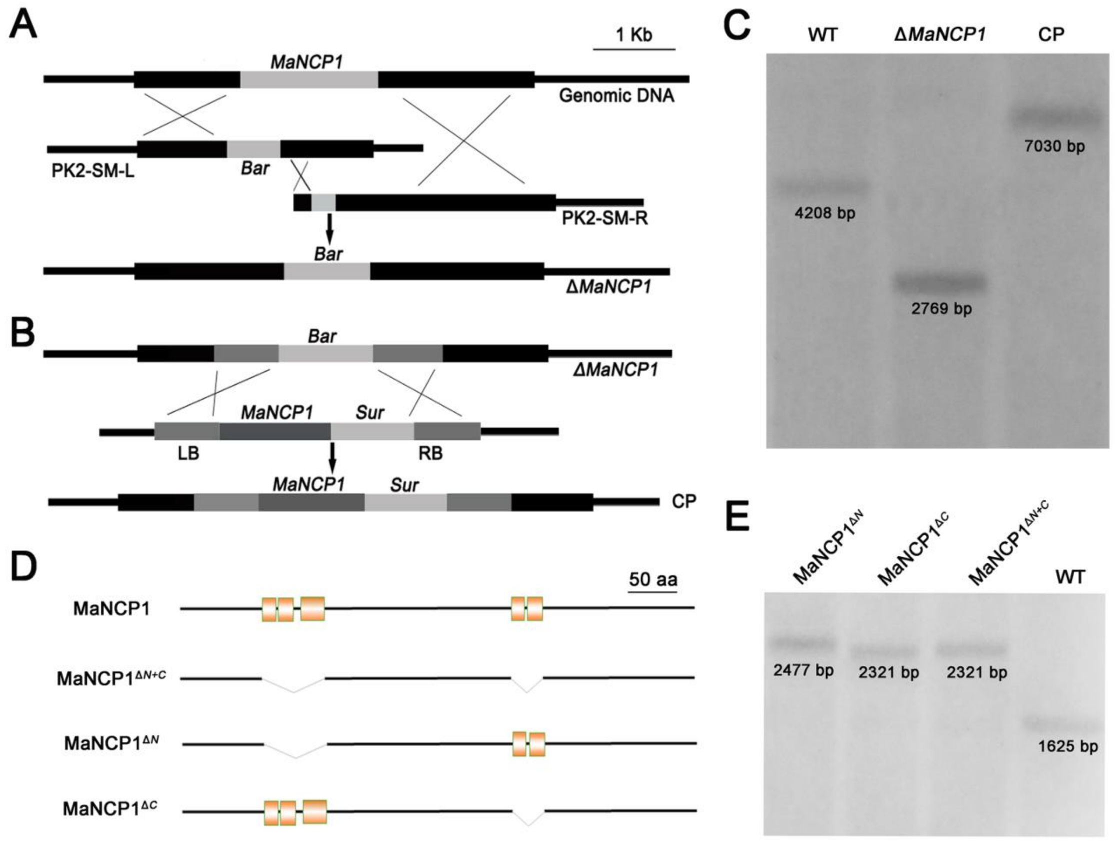

Fig. S2 Mutant constructions and Southern blotting verification. Schematic diagram of gene deletion (A) and complementation (B). (C) Southern blotting of the WT, Δ*MaNCP1* and CP strains. The probe was amplified with primers MaNCP1-PF/MaNCP1-PR (Table S3). About 6 μg of the genomic DNA from each fungal strain was digested by restriction enzyme *Pst*I for Southern blotting. The fragments of the WT, deletion and complementary strains were about 4.2 kb, 2.8 kb and 7.0 kb, respectively. (D) Schematic diagram of MaNCP1 domain deletion. (E) Verification of the WT, MaNCP1$^{\Delta N}$, MaNCP1$^{\Delta C}$ and MaNCP1$^{\Delta N+C}$ strains by Southern blotting. The probe was amplified with primers Mut-PF/Mut-PR (Table S3), and the genomic DNA was digested by *Sma*I/*Pst*I for Southern blotting. The fragments of the WT, MaNCP1$^{\Delta N}$, MaNCP1$^{\Delta C}$ and MaNCP1$^{\Delta N+C}$ strains were about 1.6 kb, 2.5 kb, 2.3 kb and 2.3 kb, respectively.

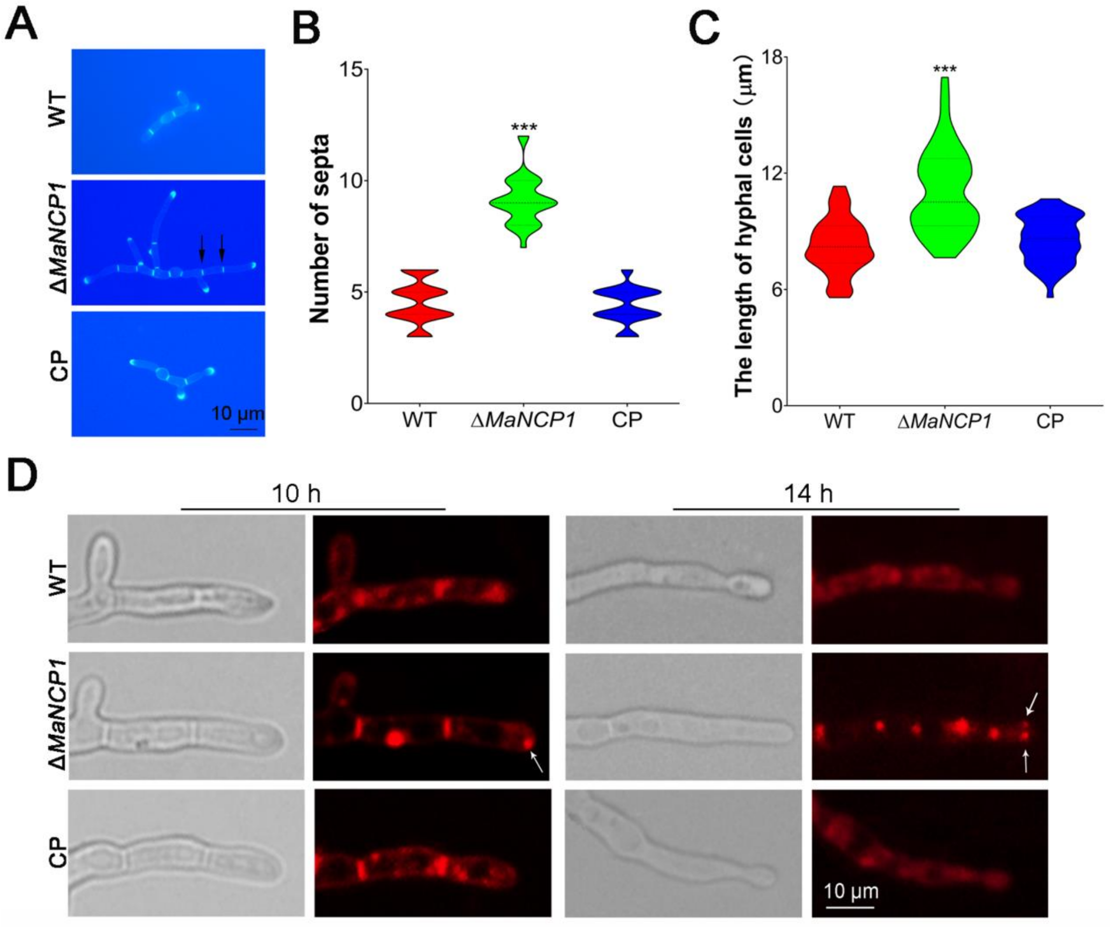

Fig. S3 Deletion of *MaNCP1* affected hyphal polar growth. (A) Hyphae of the WT, Δ*MaNCP1* and CP strains stained with CFW after 14 h of culture on SYA medium. The black arrows indicate the septa. (B) The number of septa. ***$p < 0.001$. (C) The length of hyphal cells. ***$p < 0.001$. (D) Spitzenkörper in hyphae of the WT, Δ*MaNCP1* and CP strains stained with FM4-64 after 10 h and 14 h of culture on SYA medium, respectively. The white arrows indicate the Spitzenkörpers.

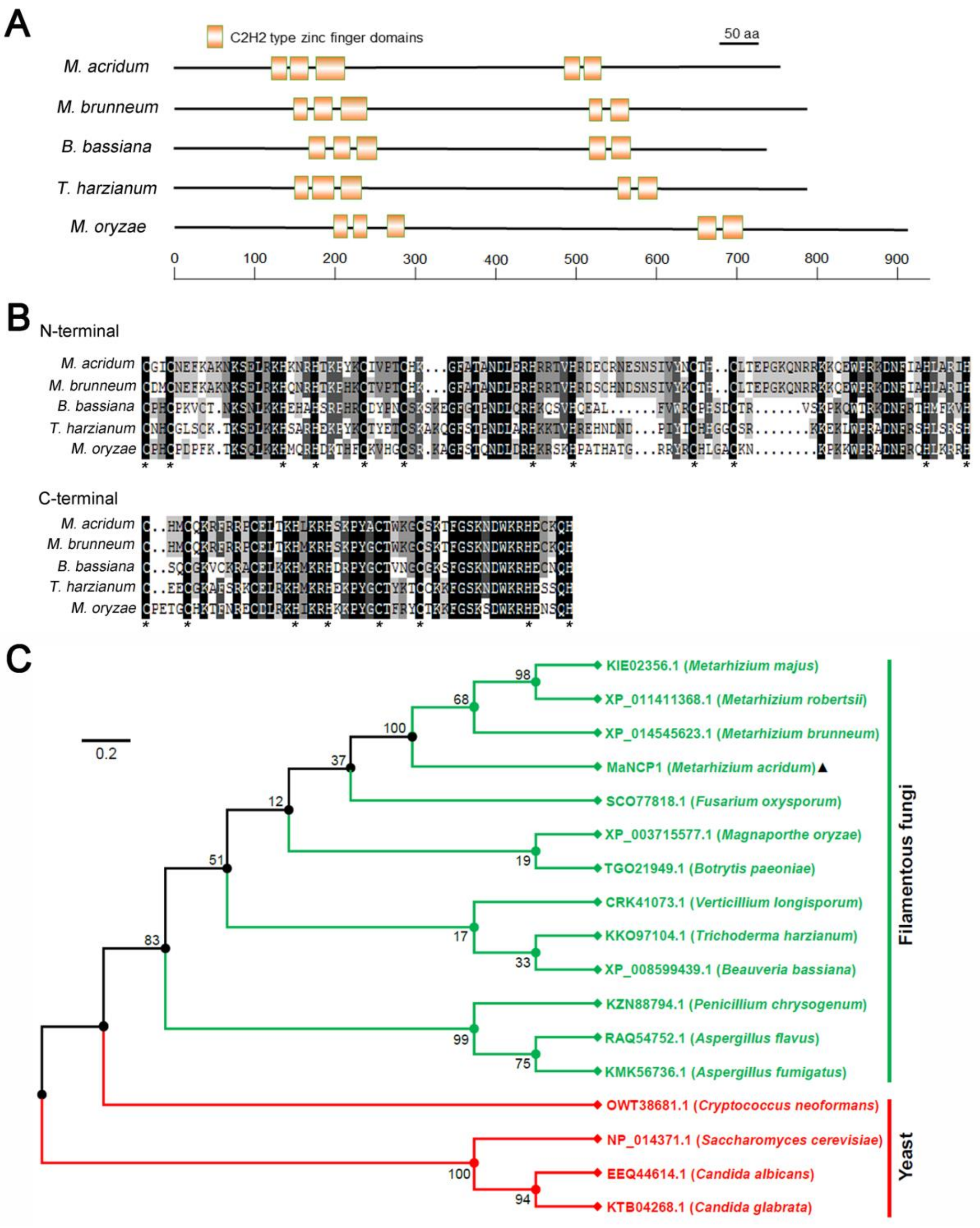

Fig. S4 Analysis of protein structure (A), C2H2 zinc fingers (B) and phylogenetic tree (C) in different fungal species. *Metarhizium acridum* CQMa102 (MAC_04326), *Metarhizium brunneum* ARSEF 3297 (XP_014545623.1), *Beauveria bassiana* ARSEF 2860 (XP_008599439.1), *Trichoderma harzianum* (KKO97104.1), *Magnaporthe oryzae* (XP_003715577.1). The asterisk represents cysteine or histidine. The black triangle represented MaNCP1 protein in *M. acridum*.

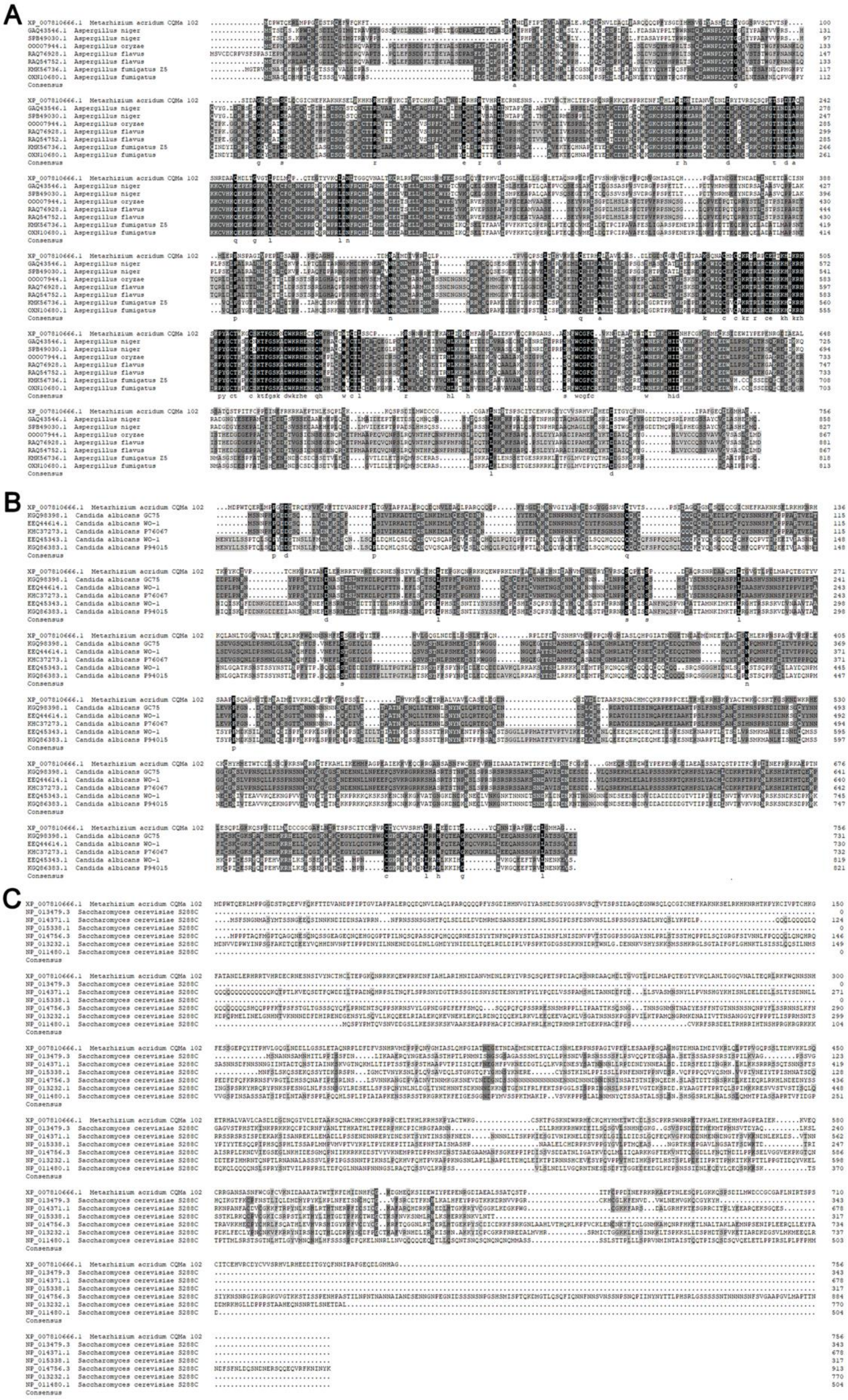

Fig. S5 Alignment analysis of the amino acid sequences among MaNCP1 and its homologous proteins from *Aspergillus* fungi (A), *Candida albicans* (B) and *Saccharomyces cerevisiae* (C).

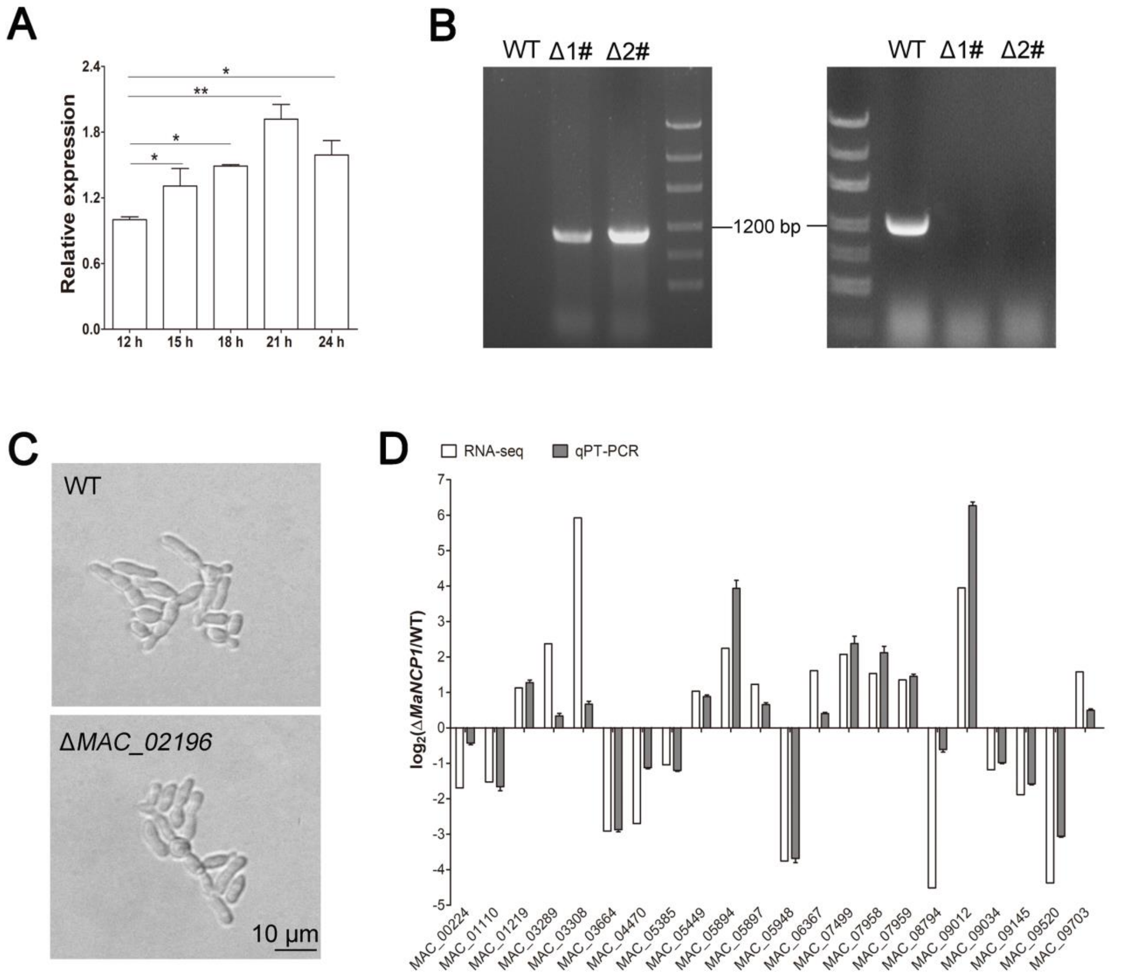

Fig. S6 The verification of the RNA-seq analysis of Δ*MaNCP1*-*vs*-WT. (A) Analysis of the expression pattern of *MaNCP1* by qRT-PCR. Samples of the WT strain were collected after growing on SYA at 28°C for 12, 15, 18, 21 and 24 h, respectively. *$p < 0.05$, **$p < 0.01$. (B) Verification of the *MAC_02196* deletion transformants. Sequences amplification of *bar* gene (left) and *MAC_02196* gene (right) in the fungal strains. WT, wild-type. Δ, the *MAC_02196* deletion transformants. (C) Conidiation pattern of the Δ*MAC_02196* strain grown on SYA. (D) The verification of the RNA-seq data by qRT-PCR.

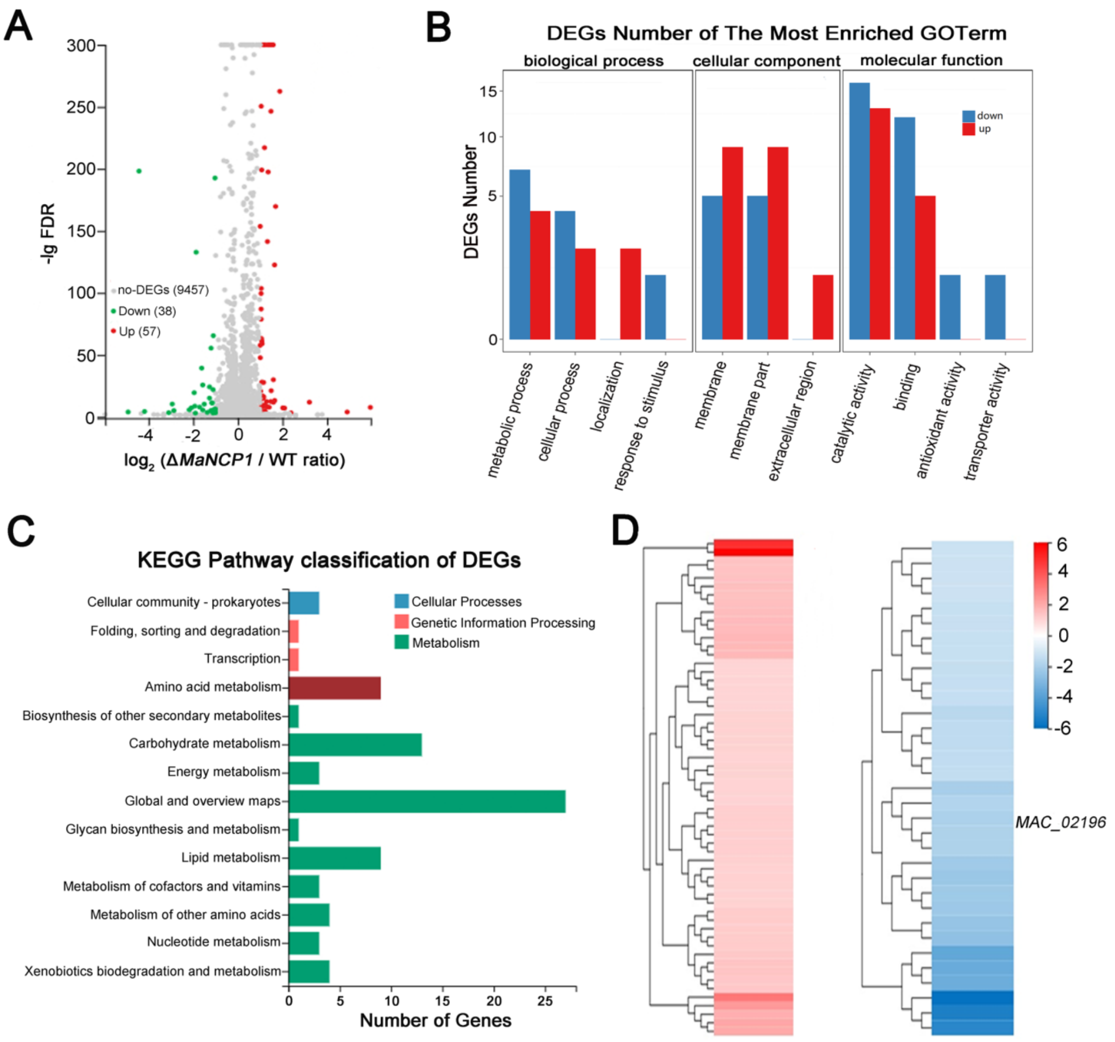

Fig. S7 RNA-seq analysis of Δ*MaNCP1* *vs.* WT. (A) Distributions of DEGs. FDR, false-discover rate. (B) The number of DEGs enriched in GO function classes. (C) KEGG pathway classification of DEGs. (D) Clustering analysis of the up-/down-regulated DEGs.

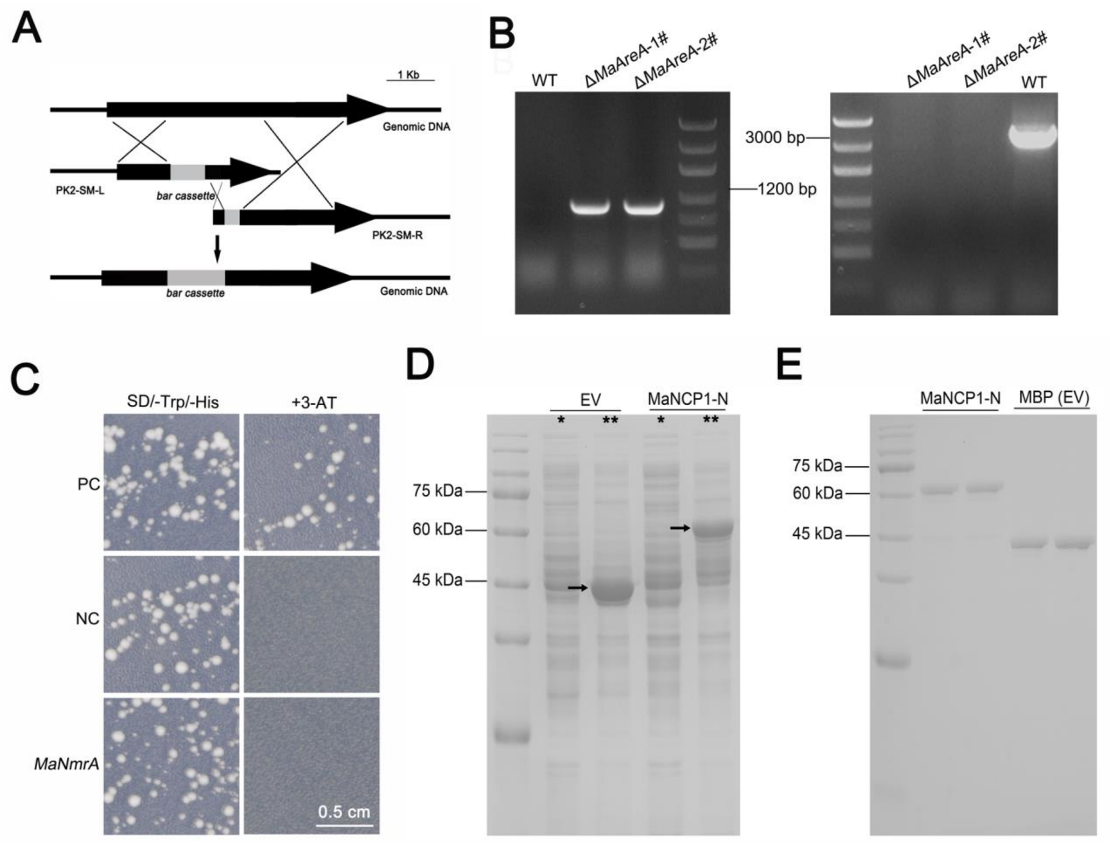

Fig. S8 Confirmation of deletion of the gene *MaAreA* and verification of target gene of MaNCP1 protein. (A) Schematic diagram of gene deletion. (B) Verification of the *MaAreA* deletion transformants. Sequences amplification of *bar* gene (left) and *MaAreA* gene (right) in the fungal strains. (C) The growth of yeast strains cultured on SD/-Trp/-His with or without 55 mM 3-AT. All plasmids were transformed into Y187 yeast strain. PC, positive control. NC, negative control. (D) The induced expression of empty vector (EV) contained the MBP-tag protein, the zinc finger cluster at the N-terminal of MaNCP1 (MaNCP1-N) in *Escherichia coli* BL21 (DE3). The arrows indicated the induced target proteins, MBP-tag protein and MaNCP1-N. *, uninduced. **, induced with 0.5 mM IPTG. (E) Purification of MaNCP1-N protein and MBP-tag protein via ÄKTA™ *prime* plus protein purification system.

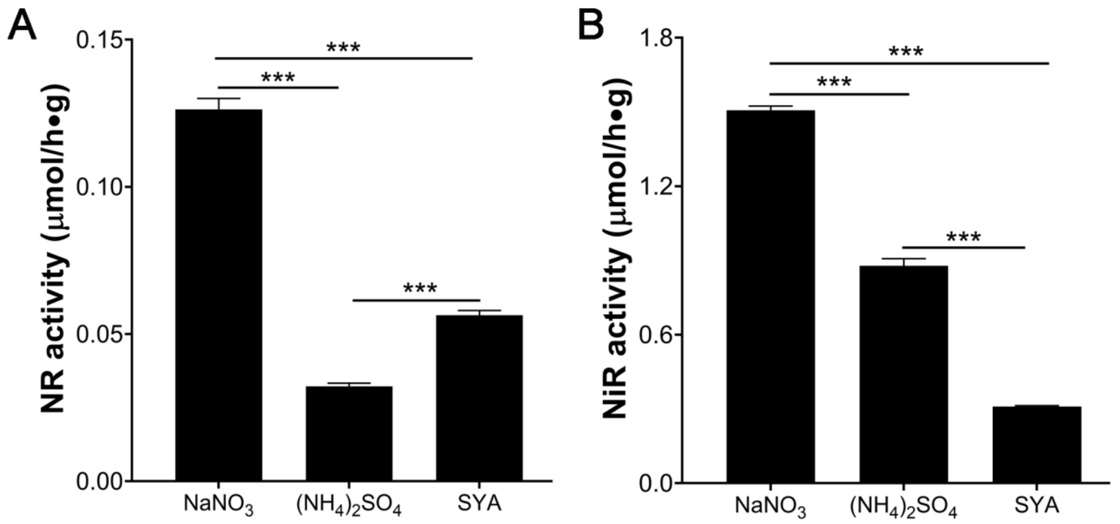

Fig. S9 Determination of the NR (A) and NiR (B) activities in the WT strain grown on SYA or modified CZA (30‰ sucrose, 1‰ K_2HPO_4, 2‰ $NaNO_3$, 0.5‰ $MgSO_4$, 0.01‰ $FeSO_4$, 0.5‰ KCl and and 18‰ agar, w/v). The nitrogen source contents in SYA ($NaNO_3$) was 30‰, were 30‰ and 15‰ in the modified CZA (nitrate) and CZA (ammonium), respectively. All fungal strains were cultured at 28 ℃ for 24 h. *** $p <$ 0.001.

Table S1 DEGs in the WT and Δ*MaNCP1* strains analysised by RNA-seq

Gene ID	log2(Δ*MaNCP1*/WT)	Regulated	Q-value	P-value	Description
MAC_03308	5.921611359	Up	1.84E-08	7.47E-09	hypothetical protein
MAC_09012	3.946623247	Up	0.000158682	0.000109264	Floculation protein FLO1
MAC_09507	3.205357516	Up	8.96E-13	2.64E-13	hydrophobin
MAC_03289	2.37214223	Up	0.000969091	0.000782949	hypothetical protein
MAC_00330	2.33894067	Up	0.000664771	0.000518801	hypothetical protein
MAC_05894	2.246183529	Up	8.59E-09	3.35E-09	oxidoreductase
MAC_07499	2.075906264	Up	1.00E-08	3.94E-09	hypothetical protein
MAC_08505	1.850939899	Up	3.54E-259	4.72E-261	hypothetical protein
MAC_02330	1.679089764	Up	1.95E-171	4.78E-173	hypothetical protein
MAC_06367	1.613007338	Up	8.86E-119	3.24E-120	hypothetical protein
MAC_09703	1.577100407	Up	4.93E-13	1.42E-13	FAD binding domain-containing protein
MAC_02361	1.560555404	Up	0	0	hypothetical protein
MAC_02602	1.538853748	Up	2.42E-29	3.33E-30	hypothetical protein
MAC_02331	1.537611251	Up	1.43E-23	2.41E-24	hypothetical protein
MAC_07958	1.531585748	Up	9.00E-12	2.82E-12	tyrosinase, putative
MAC_02717	1.487415443	Up	0	0	tyrosinase 2
MAC_08541	1.474288476	Up	1.68E-244	2.51E-246	hypothetical protein
MAC_01451	1.470185203	Up	2.15E-13	6.05E-14	NlpC/P60-like cell-wall peptidase
MAC_08917	1.415176256	Up	1.12E-08	4.44E-09	UDP-glucose 4-epimerase, putative
MAC_04929	1.38413179	Up	0	0	hypothetical protein
MAC_03674	1.351543529	Up	1.46E-197	2.85E-199	hypothetical protein
MAC_07959	1.35049442	Up	0	0	hydantoinase/oxoprolinase, putative
MAC_01123	1.302103902	Up	4.72E-06	2.51E-06	hypothetical protein
MAC_09677	1.30084088	Up	6.10E-138	1.92E-139	ferrichrome-type siderophore transporter

Table S1 (Continued)

MAC_05414	1.263295617	Up	1.95E-08	7.96E-09	short-chain dehydrogenase, putative
MAC_02992	1.242079131	Up	3.93E-19	8.02E-20	hypothetical protein
MAC_05621	1.240122934	Up	1.77E-11	5.68E-12	hypothetical protein
MAC_05897	1.230416213	Up	0.000406766	0.000302569	hypothetical protein
MAC_04278	1.213422026	Up	0	0	hypothetical protein
MAC_08520	1.199389317	Up	4.60E-12	1.42E-12	hypothetical protein
MAC_06380	1.199010409	Up	4.57E-09	1.73E-09	hypothetical protein
MAC_07769	1.193106477	Up	4.25E-31	5.51E-32	hypothetical protein
MAC_03116	1.182376047	Up	1.11E-213	1.86E-215	hypothetical protein
MAC_01219	1.129487304	Up	5.59E-05	3.57E-05	glycoside hydrolase family 24 protein
MAC_05057	1.129277265	Up	0	0	hypothetical protein
MAC_06378	1.114350693	Up	2.53E-15	6.46E-16	hypothetical protein
MAC_03878	1.094721886	Up	2.52E-18	5.33E-19	hypothetical protein
MAC_05217	1.094521942	Up	2.23E-09	8.23E-10	putative finger protein AZF1
MAC_06932	1.089581201	Up	1.14E-05	6.46E-06	hypothetical protein
MAC_07186	1.070611964	Up	7.79E-63	4.95E-64	hypothetical protein
MAC_08687	1.070137784	Up	6.53E-17	1.51E-17	arylsulfatase precursor
MAC_04515	1.059024547	Up	2.01E-106	7.90E-108	hypothetical protein
MAC_06010	1.058131562	Up	8.49E-11	2.83E-11	hypothetical protein
MAC_04005	1.052613943	Up	1.26E-57	8.94E-59	hypothetical protein
MAC_09197	1.043744089	Up	7.70E-193	1.62E-194	beta-1,6-glucanase
MAC_06018	1.04178048	Up	1.07E-06	5.29E-07	Major Facilitator /Superfamily protein
MAC_00287	1.037658323	Up	1.80E-22	3.17E-23	penicillin-binding protein, putative
MAC_05449	1.035202428	Up	0	0	epoxide hydrolase 1
MAC_01838	1.032752523	Up	8.32E-63	5.31E-64	hypothetical protein

Table S1 (Continued)

MAC_07763	1.031965709	Up	6.76E-247	9.57E-249	hypothetical protein
MAC_03905	1.02761713	Up	3.87E-86	1.81E-87	integral membrane protein
MAC_08845	1.024625759	Up	7.98E-27	1.19E-27	hypothetical protein
MAC_01616	1.019976999	Up	2.17E-97	9.14E-99	hypothetical protein
MAC_03407	1.018443111	Up	3.79E-76	2.03E-77	hypothetical protein
MAC_06062	1.01479475	Up	4.59E-06	2.44E-06	integral membrane protein
MAC_05908	1.007566011	Up	3.34E-16	8.01E-17	hypothetical protein
MAC_06958	1.001764801	Up	8.68E-13	2.55E-13	IQ calmodulin-binding motif protein
MAC_07534	-2.364113201	Down	0.000306379	0.000222581	hypothetical protein
MAC_02806	-1.0092542	Down	2.29E-07	1.04E-07	RNA polymerase II mediator complex component Srb8, putative
MAC_00301	-1.017954699	Down	2.54E-21	4.70E-22	C2 domain protein
MAC_05270	-1.035320259	Down	6.37E-17	1.47E-17	ferric reductase transmembrane component, putative
MAC_02832	-1.041066186	Down	8.74E-62	5.74E-63	hypothetical protein
MAC_05385	-1.041484442	Down	2.52E-209	4.44E-211	conidial pigment polyketide synthase PksP/Alb1
MAC_00595	-1.134296748	Down	6.62E-24	1.10E-24	cytochrome P450, putative
MAC_04111	-1.143574562	Down	0.000230488	0.000163262	hypothetical protein
MAC_04469	-1.149378573	Down	3.71E-12	1.14E-12	NADH-cytochrome B5 reductase, putative
MAC_06945	-1.161709375	Down	1.94E-12	5.85E-13	P450 monooxygenase
MAC_09034	-1.18216306	Down	1.33E-54	1.02E-55	protein phosphatase regulatory subunit Gac1
MAC_02437	-1.200218141	Down	6.10E-06	3.30E-06	ankyrin repeat and SAM domain containing protein 6
MAC_09143	-1.202240139	Down	2.09E-06	1.08E-06	putative trehalose-6-phosphate synthase/trehalose phosphatase
MAC_05266	-1.212922609	Down	2.28E-15	5.78E-16	hypothetical protein
MAC_06176	-1.25982763	Down	0.000151508	0.000104041	hypothetical protein
MAC_04978	-1.297018364	Down	0.000125371	8.47E-05	short-chain dehydrogenases/reductase, putative
MAC_00302	-1.32510586	Down	1.66E-28	2.36E-29	C2 domain protein

Table S1 (Continued)

MAC_09548	-1.409520563	Down	2.10E-05	1.24E-05	phytanoyl-CoA dioxygenase
MAC_03476	-1.510090652	Down	1.24E-05	7.09E-06	coatamer subunit protein
MAC_01110	-1.523180056	Down	2.00E-26	3.03E-27	cytochrome P450, putative
MAC_04876	-1.53142405	Down	0.000384447	0.000284942	hypothetical protein
MAC_02196	-1.582629821	Down	4.91E-40	4.98E-41	NmrA family transcriptional regulator
MAC_05689	-1.605917776	Down	3.95E-08	1.68E-08	sulfatase domain protein
MAC_00224	-1.694922782	Down	2.18E-14	5.83E-15	GAL4 & Fungal specific transcription factor domain containing protein
MAC_05976	-1.819733197	Down	2.42E-132	7.96E-134	hypothetical protein
MAC_02266	-1.883451751	Down	1.66E-07	7.51E-08	hypothetical protein
MAC_09145	-1.887366801	Down	2.90E-09	1.08E-09	carboxylesterase family protein
MAC_00141	-1.887698723	Down	1.05E-19	2.10E-20	hypothetical protein
MAC_01780	-2.190880277	Down	0.000649151	0.000505398	hypothetical protein
MAC_03232	-2.223301754	Down	1.05E-06	5.18E-07	hypothetical protein
MAC_04470	-2.697232943	Down	4.64E-05	2.90E-05	catalase
MAC_03664	-2.911357748	Down	1.04E-10	3.51E-11	hypothetical protein
MAC_06701	-3.375304848	Down	5.01E-05	3.17E-05	extracellular serine-rich protein
MAC_05948	-3.753816471	Down	0.000616351	0.000476076	hypothetical protein
MAC_05687	-3.860731675	Down	0.000342922	0.00025165	nonribosomal peptide synthase
MAC_09520	-4.375304848	Down	1.02E-05	5.75E-06	amino acid transporter, putative
MAC_04326	-4.463162353	Down	1.29E-206	2.38E-208	C2H2 type zinc finger domain protein
MAC_08794	-4.512808372	Down	0.000882493	0.000705209	hypothetical protein

Table S2 Primer combinations used in fusion PCR

First round PCR	Second round PCR	Third round PCR	Template	Mutant
DD-LF/DD-13R	DD-13F/DD-LR	DD-LF/DD-LR	cDNA	ΔN
DD-LF/DD-M3R	DD-M3F/DD-LR	DD-LF/DD-LR	cDNA	ΔC
DD-LF/DD-M3R	DD-M3F/DD-LR	DD-LF/DD-LR	ΔN	ΔN+C

Table S3 Primers used in this study

Primer	Sequence (5'-3')	Description
Bar-F	GCTCTACACCCACCTGCT	Universal primers of the gene deletion vector
Pt-R	CAGCCAAGCCCAAAAAGTG	
Bar-F1	GAAGAATGACATTGAAGGAGC	For the sequences amplification of *Bar* gene
Bar-R1	CCCGTCACCGAGATCTAATAA	
M13F	TGTAAAACGACGGCCAGT	Universal primers for cloning vector
M13R	CAGGAAACAGCTATGACC	
MAC_02692-LF	GACGGCCAGTGCCAAGCTTTCAACTTTGACGCAGACCA	For *MAC_02692* deletion vector construction and transformants verification.LF/LR, RF/RR were used for PCR amplification the left and right border sequences, respectively. VF/Pt-R, Bar-F/VR were used for PCR verification the left and right border, respectively.
MAC_02692-LR	CGGATCCCTCGAGTCTAGCTAAAAGGGTTGCGGGTTTT	
MAC_02692-RF	ACCGAGATCTAATAAGATTGGGCTCCTTGCTGTTTAAG	
MAC_02692-RR	ATGACATGATTACGAATTAGAAGTGACGAACCGACTGG	
MAC_02692-VF	CACCGTCACGATCGAATACA	
MAC_02692-VR	TGACATCCGGTCACTCAAGA	
MAC_04326-LF	GACGGCCAGTGCCAAGCTGCAGACAAACATGCAAGCAC	For *MAC_04326* deletion vector construction and transformants verification
MAC_04326-LR	CGGATCCCTCGAGTCTAGATACAGGCAGCGGTGAGAAG	
MAC_04326-RF	ACCGAGATCTAATAAGATTAAGCACGCTCCTTTGATCC	
MAC_04326-RR	ATGACATGATTACGAATTGTCCCAATGCCATCTCAAAC	
MAC_04326-VF	GCACCACCTCCACTTCTCAT	
MAC_04326-VR	CAGCCCAGGGAGTTGAATTA	
MAC_03846-LF	GACGGCCAGTGCCAAGCTCACGACGCTCACGAGATATG	For *MAC_03846* deletion vector construction and transformants verification
MAC_03846-LR	CGGATCCCTCGAGTCTAGATCCCCAGTTGTCACCAGAA	
MAC_03846-RF	ACCGAGATCTAATAAGATAACCACTTGTTGCCGATGAT	
MAC_03846-RR	ATGACATGATTACGAATTGGTCGTCTCGAGGAGTTTGA	
MAC_03846-VF	GGAGGCTCGTAACCTCACCT	
MAC_03846-VR	CTGAGGAAGGGGGATGAGAT	

Table S3 (Continued)

MAC_02196-LF	GACGGCCAGTGCCAAGCTTACATGGTGATAGCGTATTG	For *MAC_02196* deletion vector construction and transformants verification
MAC_02196-LR	CGGATCCCTCGAGTCTAGTTGAGTTCTCACAAGGTCAT	
MAC_02196-RF	ACCGAGATCTAATAAGATTTTGTGGAAGAGAACAAGGC	
MAC_02196-RR	ATGACATGATTACGAATTGAATGGAATTCCGCCATTCA	
MAC_02196-VF	CGTATAACCTTGACATCAGT	
MAC_02196-VR	ATCTTCTCATCATGGAGGTG	
MAC_02196-F	ATGTCCAAGGTCTTTACTGTT	For the sequences amplification of *MAC_02196*
MAC_02196-R	TTAATCCCACTTGGCCTTGTT	
AreA-F	ATGCCCATCCGCCTCGCGACG	For the sequences amplification of *MaAreA*
AreA-R	TTAAAGACTCATTGTTAACCAT	
AreA-LF	GACGGCCAGTGCCAAGCTATGGGCAACACTGACAACA	For *MaAreA* knockout vector construction and transformants verification
AreA-LR	CGGATCCCTCGAGTCTAGGCAGTCGGTCACTCATTCA	
AreA-RF	ACCGAGATCTAATAAGATCACAGCGGCAGGCTATTT	
AreA-RR	ATGACATGATTACGAATTCGGCTCAATGATGGGATG	
AreA-VF	TTATCCATCTCTCGTCCAACGC	
AreA-VR	TAGAACGAGCCGTGGAGTCCTA	
CP-LF	GACGGCCAGTGCCAAGCTTGCAGACAAACATGCAAGCACTA	For *MaNCP1*complementation vector construction and transformants verification
CP-LR	GGGATCCCTCGAGTCTAGATAGGACCCAGTGGAAAGGCCAC	
CP-RF	GCTGGCCGCCCATGGGATATCGTCGACGTGAGAGCATGCAATT	
CP-RR	CTATGACATGATTACGAATTCTCAGTCGACGTGCCAACGCCAC	
CP-VF	TCAGACGATGACAATTGAAG	
CP-VR	ACTGGTCCCTCAAATATGC	
MaNCP1-PF	GCAGACAAACATGCAAGCAC	For amplification probe that used in southern blotting of WT, Δ*MaNCP1* and CP strains
MaNCP1-PR	AGGATGCAGGTGCGAGATG	

Table S3 (Continued)

DD-LF/Mut-LF	GACGGCCAGTGCCAAGCTATGGACCCGTGGACTCAGGAGCG	For MaNCP1 domain deletion mutants construction and transformants verification
DD-LR	CGGATCCCTCGAGTCTAGTCATCCCGCATGCATGCCCAGA	
DD-RF	ACCGAGATCTAATAAGATGACTCGAAATTCACGGCGGAT	
DD-RR	ATGACATGATTACGAATTCAGCAAAATGATGGACAACGG	
DD-13F	CAATTGGTCCCAAAATATTGACGCCA	
DD-13R	TGGCGTCAATATTTTGGGACCAATTG	
DD-M3F	CCAAGAGTCAGTATCATATGGA	
DD-M3R	TCCATATGATACTGACTCTTGG	
Mut-LR	CGGATCCCTCGAGTCTAGGGGGCTCCCCAGAGGACTCAAA	
DD-VF	ATGCAACCTGGCATTCGAAA	
DD-VR	TGAGACTGTCACAACATGTA	
Mut-PF	GCGATTTGAGCTCTTGCC	For amplification probe that used in southern blotting of domain deletion mutants
Mut-PR	TTCCAAGCGGCTGGGACT	
MaNCP1-OF	ATTTCCCCTAAGTACTTCTAGAATGGACCCGTGGACTCAGGAG	For *MaNCP1* overexpression vector construction with NCP1-OF/NCP1-OR and transformants verification with NCP1-OF/GFP-VR.
MaNCP1-OR	CCTTGCTCACCATGGATCCTCCCGCATGCATGCCCAGATC	
GFP-VR	CGATGCGGTTCACCAGGGTGT	
NmrA-OF	TTATTTCCCCTAAGTACTACTATGCCAGCCCAAATTACCG	For MaNmrA overexpression vector construction with NmrA-OF/NmrA-OR and transformants verification with NmrA-OF/Nat-VR.
NmrA-OR	GAGGCCATCTCGAGACTAGTTGCCAGCCATTGTTCTTCGT	
Nat-VR	AAATGTACGGGCGACAGT	
MaNCP1-F	ATGGACCCGTGGACTCAGGAG	For the cDNA sequences amplification
MaNCP1-R	TCATCCCGCATGCATGCCCAG	
NCP1-pCold-F	GAAAACCTGTATTTTCAGTCCATGGACCCGTGGACTCAGGAG	Fpr prokaryotic expression vector construction
NCP1-pCold-R-N	CAGGTCGACAAGCTTGAATTCTCAACGAACAATATATCGATCC	

Table S3 (Continued)

MaNCP1-BD-F	GAGGAGGACCTGCATATGATGGACCCGTGGACTCAGGAGC	For yeast expression vector construction. pHIS2-F/R, the universal primers of pHIS2 vector. T7/3' BD and T7/3' AD are the the universal primers of pGBKT7 and pGADT7 vector, respectively.
MaNCP1-BD-R	CTGCAGGTCGACGGATCCTCATCCCGCATGCATGCCCAGA	
MaNCP1-AD-F	TGGCCATGGAGGCCAGTGATGGACCCGTGGACTCAGGAGC	
MaNCP1-AD-R	GCTCGAGCTCGATGGATCTCATCCCGCATGCATGCCCAGA	
pHIS2-NmrA-F	GACTCACTATAGGGCGAATTCTCTAGGTTCTTTAGGTTAGTCG	
pHIS2-NmrA-R	ATAATGCCAGGAATTACTAGTTGTTACTCTGGTCGTTGCCAAT	
pHIS2-AreA-F	GACTCACTATAGGGCGAATTCAACGAGTCCAAGCAAGTAATGA	
pHIS2-AreA-R	ATAATGCCAGGAATTACTAGTGGACATTTTCGTCCATGCAGT	
pHIS2-F	TGCGGGCCTCTTCGCTATTAC	
pHIS2-R	AGGGCTTTCTGCTCTGTCATC	
T7	TAATACGACTCACTATAGGGCG	
3' BD	TTTTCGTTTTAAAACCTAAGAGTC	
3' AD	AGATGGTGCACGATGCACAG	
Pbobe-F	CACTTGAGCTCTTATCAATT	For the EMSA probe amplification
Pbobe-R	AAAGATCAATAGGAGCTCAGA	
gpdh-qF	GACTGCCCGCATTGAGAAG	For qRT-PCR assay
gpdh-qR	AGATGGAGGAGTGGGTGTTG	
MAC_00186-qF	GTAGACGAGGCTGTTAGG	For qRT-PCR assay
MAC_00186-qR	ACGACTAGGATTATGAGATTGA	
MAC_08242-qF	AAGGAGGAGCGAAGATTC	
MAC_08242-qR	GCGTATATTAAGCCACACA	
MAC_02692-qF	GGTATCAGCAAGAAGGATT	
MAC_02692-qR	CGACATTGAGTTGGTGAA	
MAC_03472-qF	TATACCGCCACTACTTCT	
MAC_03472-qR	ATACCTCCTCAACATCCT	

Table S3 (Continued)

MAC_04326-qF	TTACTTGGCTCTTCATTG	
MAC_04326-qR	GTCGGTCTCATCATTATC	
MAC_06473-qF	CGTATCATTTCGTTCTTT	
MAC_06473-qR	TGACGGTATAACTAATCC	
MAC_03846-qF	CCAGGAAGGTCACATAGC	
MAC_03846-qR	TCATCATCGGCAACAAGT	
MAC_03700-qF	AACAAAGGAATCTGAGAAAG	
MAC_03700-qR	CTGATTGTGACGGAGTAT	
AreA-qF	TATTGATGAGCGTCGTAA	
AreA-qR	ATCGTAACTGAATTGGTATT	
AreB-qF	CAAATAGAGCAACCTGAAT	
AreB-qR	CTCGTTGATAGTCTCCTC	
NmrA-qF	CATCTATAACAACAACTTC	For qRT-PCR assay
NmrA-qR	CGTCTTTGAATAACTGTA	
Fhb1-qF	CATTGTCGCTTCCACTAG	
Fhb1-qR	TAATACTCTGCCGTCTTG	
Fhb2-qF	GTCAACACATTCGTTCAG	
Fhb2-qR	CCAGTCTATTCAAGTCCAT	
NrtB-qF	CGTAGTTGATCCGTATGG	
NrtB-qR	TAGAAGTAGAGTAGCACAGA	
NR-qF	AATGGTTGAAGAGGATAA	
NR-qR	GTAACAGATGACACTATTG	
NiR-qF	GTATCAACACCAAAGTCA	
NiR-qR	AATCGTCAATGGTTCTATA	
GOGAT-qF	AAAGTTTGACGAGAGATTG	

Table S3 (Continued)

GOGAT-qR	GTAGTATGAGTAGACCTGAA	
GS1-qF	GCACAGTAACTTCTCAAC	
GS1-qR	TCATTGTCCTCTCCATAC	
GS2-qF	TTTATGAGAAAGGATAGCA	
GS2-qR	TTAGACGGTTTCAATGTA	
GS3-qF	CACCTCCATCTTTCTACT	
GS3-qR	GAACATAGCCGTCATAGT	
GDH1-qF	TACTGTCAACCTGTCCAT	
GDH1-qR	ATTTCGCCATCAGACTTG	
GDH2-qF	GTATCCGTATTGTCAAGT	
GDH2-qR	GGTCAAGAAGAATAACAC	
Ape1-qF	CGAATACAATGGAACAAC	
Ape1-qR	GACGATATGGTTAGGAAG	For qRT-PCR assay
Ape2-qF	ATGAGATTGGCGGTGATT	
Ape2-qR	ATGATGAGATGATGATGAAGG	
MAC_00224-qF	ATGAGACTACCAATAGAATC	
MAC_00224-qR	TCGGGAGCAATAATAATG	
MAC_01110-qF	TTGAAGTTTACAGAATGG	
MAC_01110-qR	CTTGGGAAATAATATCGTA	
MAC_01219-qF	GAGATTCACACCATTACG	
MAC_01219-qR	AAATCTTTGTGGGAACTT	
MAC_03289-qF	AGACAGAATTAAAGACAAC	
MAC_03289-qR	AATAAGACTGGAACACAT	
MAC_03308-qF	GAGAATGTTCGTGAATATC	
MAC_03308-qR	TACTGATTGCTGTTCTTT	

Table S3 (Continued)

MAC_03664-qF	TACTTTATGGCTGATTAC	
MAC_03664-qR	GGTAGAGAGTATTGTATAG	
MAC_04470-qF	GGGAATGTGACTTGCTTT	
MAC_04470-qR	GATTATGCTATGAAGGAGTTGT	
MAC_05385-qF	TGGACTCATCACAAGAAG	
MAC_05385-qR	GCTGTGGTTGTATAAGAC	
MAC_05449-qF	AGAATGCCAAGAATACCT	
MAC_05449-qR	GAGAAGAGAGCCAGAAAG	
MAC_05894-qF	AAGAATATAGAATTGAGAGG	
MAC_05894-qR	CTTCAAGTCTTCAAACAA	
MAC_05897-qF	TGACAATGGCATAACTCT	
MAC_05897-qR	ATGAATGGTGTGTTGATG	
MAC_05948-qF	ATTGAGACGAACCATTAC	For qRT-PCR assay
MAC_05948-qR	AAACACCAACAAGAAACT	
MAC_06367-qF	GCTCCCAGAACATCATTT	
MAC_06367-qR	CGAGGTGAATAAGAAGGA	
MAC_07499-qF	CGGTGTATCTTTGAATCTC	
MAC_07499-qR	ATTTCCTTGCCTTGTTTC	
MAC_07958-qF	ATCCAGCACATCCAGTAT	
MAC_07958-qR	TAGGAGTTTGGGTGGTAG	
MAC_07959-qF	AAGACGATGCTATTGTAT	
MAC_07959-qR	GTCAAATCTCTCTATATGC	
MAC_08794-qF	AGTATTCTATGCTCTCTG	
MAC_08794-qR	AATAATAGTCTCTTCGTC	
MAC_09012-qF	ACTGGAGGCAATTATATC	

Table S3 (Continued)

MAC_09012-qR	GTCTTGATAGTGAGGTTA	For qRT-PCR assay
MAC_09034-qF	ATTCTGTATTCGCTATCG	
MAC_09034-qR	GGAGCATCTGTACTATTC	
MAC_09145-qF	GATTCCTTCCATACTTCTCT	
MAC_09145-qR	TCGTCCTCGTTATTGATG	
MAC_09520-qF	TAAGTGGATTCTGATGGT	
MAC_09520-qR	TTATGCTGAGATGGAGTT	
MAC_09703-qF	GTCATTACCTCTATTACG	
MAC_09703-qR	TGAGATACCAAATAAGTG	
